# Biomechanical mechanisms underlying the effect of minimalist footwear on walking stability in persons with a history of falls

**DOI:** 10.1038/s43856-025-01291-x

**Published:** 2025-12-16

**Authors:** Tomasz Cudejko, Asangaedem Akpan, Kristiaan D’Août

**Affiliations:** 1 Department of Sport, Exercise and Rehabilitation, Northumbria University, Newcastle upon Tune, UK; 2 Bunbury Regional Hospital, Western Australia Country Health Service South West, Bunbury, WA Australia; 3 Division of Internal Medicine, University of Western Australia, Crawley, WA Australia; 4 Medical School, Faculty of Health, Curtin University, Bentley, WA Australia; 5 Department of Musculoskeletal and Ageing Science, Institute of Life Course and Medical Sciences, University of Liverpool, Liverpool, UK

**Keywords:** Lifestyle modification, Motor control, Ageing

## Abstract

**Background.:**

Footwear design influences sensory input and motor control during gait—key factors in fall risk among older adults. Our previous work showed that minimalist footwear alters walking stability in individuals with a history of falls, but the underlying biomechanical mechanisms remain unclear. Here, we investigated how footwear type influences lower-limb biomechanics, whether these effects are altered by cognitive load, and whether they mediate adaptations in gait stability.

**Methods.:**

In this cross-sectional repeated-measures design, thirty older adults with a history of falls (mean ± SD age 68.6 ± 4.4 years) completed walking trials under three footwear types (barefoot, supportive, minimalist) and two task conditions (single and dual-task with cognitive load). 3D kinematics, ground reaction forces, and surface electromyography were collected to quantify joint angles, powers, and muscle activity. Statistical parametric mapping and linear mixed models tested condition effects and cognitive load interactions, while mediation analysis assessed whether biomechanical changes explain previously reported stability differences.

**Results.:**

Here we show that minimalist shoes induce distinct biomechanical adaptations, including greater ankle dorsiflexion and external rotation, reduced hip flexion during stance, and increased knee flexion during swing. They also enhance hip and ankle joint power generation and elicit higher activity in rectus femoris and vastus lateralis. These effects are consistent across cognitive conditions. Hip kinematics, kinetics, and quadriceps muscle activity mediated adaptations in gait stability observed with minimalist footwear.

**Conclusions.:**

These findings identify specific neuromuscular changes associated with minimalist footwear that may explain adaptations in walking stability. Our results support the development of footwear-based interventions for fall prevention in older adults and highlights the need for randomized controlled trials in broader populations to confirm causality.

## Plain language summary

Because footwear affects movement control, it may influence fall risk - a major concern for older adults. Our study examined how minimalist shoes, which are lightweight with thin, flexible soles. supportive shoes, and barefoot walking change leg movements and muscle activity in older adults with a history of falls. We measured 30 participants while they walked normally and while performing a thinking task. We found that minimalist shoes promoted a distinct walking pattern, increasing muscle activity in the thighs and changing movement dynamics. This suggests the body adopts a more active, controlled strategy to manage different sensory feedback from minimalist shoes. These findings show how footwear can alter gait mechanics in fall-prone older adults. Understanding these adaptations is a crucial step for future research into designing footwear and interventions that may help prevent falls.

## Abstract (web summary)

Cudejko et al. explore the neuromechanical mechanisms by which footwear influences walking stability in fall-prone older adults. Findings reveal that minimalist footwear induces joint and muscle biomechanical changes that explain adaptations in dynamic balance control, reflecting a shift towards a more neuromechanically engaged walking pattern.

## Introduction

The human foot constitutes the fundamental functional unit for the transmission of sensory information to the brain during walking^1^. The sensory receptors in the plantar skin and fascia, ligaments, joint capsules, muscles and tendons provide immediate sensory information about changes in foot pressure, vibration and stretch. This contributes to the modulation of the muscle reflexes during walking as well as assistance from the proprioceptive afferents in the planning and correction of movement^2^. Accurate and timely delivery of such sensory feedback is essential for controlling stability during walking and therefore may be important in the prevention of falls—a significant health and economic challenge worldwide^3^.

Modern supportive footwear, characterized by features such as cushioning, arch supports, restrictive toe boxes, and elevated heels, may dampen the sensory input provided by the foot’s receptors. These features can reduce the foot’s capacity to perceive and respond to changes in pressure, shape, and surface texture, deviating from the natural sensory experience of walking barefoot^4^. In older adults, age-related declines in muscle strength and sensory function may exacerbate this limitation, potentially increasing susceptibility to falls. While direct evidence linking footwear type to fall risk remains sparse^5,6^, research suggests that wearing supportive footwear significantly affects human mobility and movement stability^7–10^, both of which are key risk factors for falls in older adults^3^.

Our prior work demonstrated that minimalist shoes—designed to replicate the natural dynamics of barefoot walking with features such as flexibility, flat soles, and minimal cushioning—induce different adaptations in walking stability in individuals with a history of falls compared to conventional supportive footwear (hear after *supportive*) or even barefoot walking^7^. We observed that this effect was not explained by between-footwear differences in spatio-temporal parameters of gait, such as speed and cadence, suggesting presence of other underlying mechanisms. Given that minimalist footwear’s features are intended to replicate the neuro-mechanical behavior of barefoot walking^11,12^, walking in minimalist shoes might have led to mechanical alternations such as changes in lower-limb kinematics/kinetics, and/or, via increased stimulation of the foot sensory receptors, to changes in efferent mechanisms, such as muscle activity.

Research on the biomechanical effects of footwear in older adults remains limited. Franklin et al.^13^ observed that walking barefoot or in minimalist shoes resulted in a decrease in tibialis anterior activity at initial stance in older adults in comparison to supportive footwear, where the tibialis anterior normally acts eccentrically to control foot lowering and absorb power during loading. No differences were observed for gastrocnemius medialis and peroneus longus. Hannigan and Pollard^14^ found that, in older females (50–70 years), walking in maximally cushioned shoes produced higher peak knee adduction moments and walking in both maximal and minimalist shoes increased knee flexion angles and vertical loading rates compared to traditional shoes. Recent systematic review suggests that findings related to footwear-induced changes in gait biomechanics are inconsistent, with most data derived from studies on younger populations^15^. As such, a comprehensive evaluation of the effects of footwear types on lower limb biomechanics in older adults, particularly those with a history of falls, is critically lacking.

In addition, an emerging and underexplored area is the potential interaction between cognitive load and footwear effects on lower-limb biomechanics. Dual-task walking—simultaneously performing a cognitive and motor task—is known to impair gait in older adults^16^. Cognitive demands may exacerbate sensory or motor impairments, altering the effects of footwear on lower-limb biomechanics. Understanding how cognitive load influences these interactions is vital for evaluating footwear in real-world contexts, where individuals frequently walk while distracted or multitasking. Finally, it remains unknown whether differences in lower-limb biomechanics induced by footwear mediate adaptations in walking stability. Identifying biomechanical mechanisms underlying changes in walking stability associated with minimalist footwear could bridge the gap between biomechanical findings and clinical interventions for fall prevention.

This study aims to fill these crucial knowledge gaps by addressing three research questions: i) Are there differences in the effects of footwear type on lower-limb kinematics, kinetics, and muscle activity in persons with a history of falls? (ii) Are these effects influenced by cognitive load? (iii) Do biomechanical differences between footwear types explain previously observed adaptations in walking stability? We hypothesized that minimalist footwear would elicit distinct differences in lower-extremity kinematics, kinetics, and muscle activity compared with supportive shoes in persons with a history of falls. By investigating these questions, this study provides novel insights into the biomechanical and neuromuscular mechanisms underlying footwear-related changes in walking stability. This research has the potential to inform clinical recommendations for fall prevention, guide the design of safer footwear, and inspire multidisciplinary collaborations between biomechanics, neuroscience, and public health. These findings could ultimately contribute to reducing the burden of falls among older adults and improving their quality of life.

This study demonstrates that minimalist footwear induces significant alterations in lower-limb biomechanics in people with a history of falls, including increased joint mobility, enhanced hip and ankle power generation, and greater activation of the rectus femoris and vastus lateralis. Mediation analysis identified that changes in hip kinematics, hip joint power, and quadriceps activity are potential mechanisms underlying the previously observed between-footwear differences in stability during walking. These neuromechanical adaptations were consistent during cognitive tasks, indicating a robust footwear effect. Collectively, these findings highlight the specific biomechanical strategies associated with minimalist footwear, providing a mechanistic rationale for its further investigation as a potential intervention. Future research is warranted to confirm these effects in more diverse populations, under real-world conditions, and to evaluate their long-term impact on fall risk.

## Methods

### Study design

**Fig. 1:**
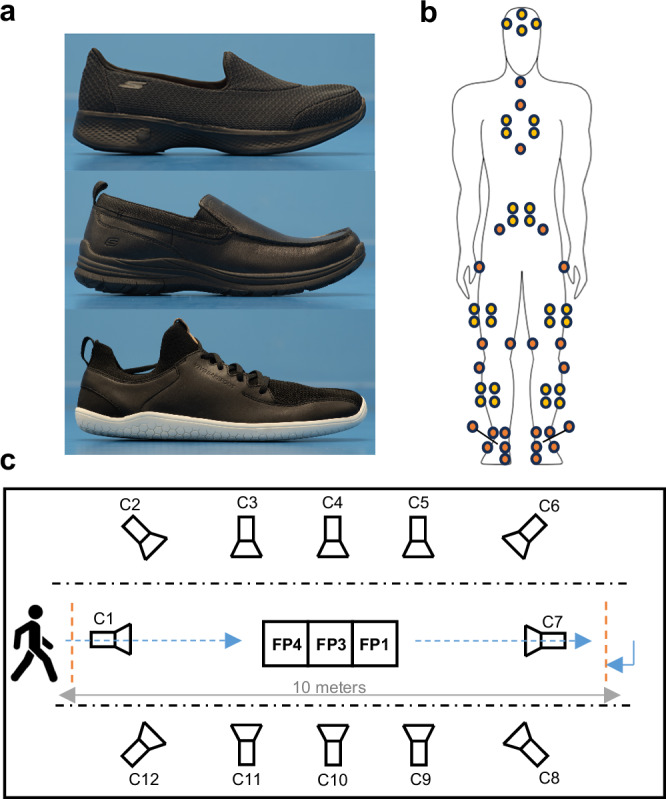
Experimental design illustrating footwear, marker configuration, and laboratory layout. **a** Shoe types assessed in this study i.e. supportive shoe for women (top) and men (middle), and minimalist shoe (bottom). **b** Location of attachment for reflective markers (marker clusters with yellow). **c** Laboratory set-up with location of the walking path and cameras.

The data presented here were collected as part of a quasi-experimental study with a repeated measures within-participant design, evaluating the effects of footwear type in persons with a history of falls. In the study, participants undertook biomechanical and mobility assessments during one laboratory visit in three footwear conditions, barefoot, supportive shoes, and minimalist shoes, in a randomized order. We tested market-available supportive shoes (Go Walk 4.0-Pursuit for females and the Superior 2.0-Jeveno shoe for males, Skechers USA, Inc.; Fig. 1a top and middle, respectively) and minimalist shoes (Primus Knit, Vivobarefoot Ltd., London, UK; Fig. 1a bottom). We used simple randomization generated from the website www.randomizer.org. Health Research Authority, Health and Care Research Wales, and East Midlands—Derby Research Ethics Committee approved the study (reference 19/EM/0197). The study protocol had been published before participant enrollment (ClinicalTrials.gov Identifier: NCT03874728). All participants signed written informed consent according to the Declaration of Helsinki before taking part. The STROBE reporting guidelines for cross-sectional observational studies were followed^17^.

### Footwear specifications

The footwear tested in this study was selected to represent two distinct categories: supportive shoes and minimalist shoes, according to the established definitions and criteria^11^. Minimalist footwear is defined by several key design principles: a thin, flexible sole that offers minimal cushioning; a ‘zero-drop’ profile where the heel height is equal to the forefoot height; a wide toe box that allows for natural toe splay; and minimal weight and motion control features. In contrast, supportive shoes typically feature a higher heel-to-toe drop, significant cushioning, structured support, and a more rigid sole. The specific models used were characterized by the following mechanical properties provided by the manufacturers: Vivobarefoot Primus Knit (minimalist shoe) has: a heel-to-toe drop of 0 mm, a stack height (sole thickness) of <4 mm, and a hardness of the outsole material of 65 Shore A. The mass of a single shoe (UK size 8) is 210 g. Skechers Go Walk 4.0 (supportive shoe for females). has a heel-to-toe drop of 12 mm, a stack height is approximately 25 mm, and hardness of the midsole foam is 55 Shore C (equivalent to approximately 75 Shore A). The mass of a single shoe (UK size 8) is 185 g. Skechers Superior 2.0 (supportive shoe for males) has a heel-to-toe drop of about 10 mm, with a heel stack height around 27–29 mm. The EVA midsole foam measures roughly 30–35 Shore C (≈45–50 Shore A). The mass of a single shoe (men’s UK 8) is ~250 g.

### Participants

We recruited persons with a history of falls from the local community via adverts in the University of Liverpool intranet, GPs, and the University of the Third Age. The inclusion criteria were age ≥ 60 years, and ≥ 1 self-reported fall after the age of 60. A fall was defined as an unintentional fall to the ground, not preceded by loss of consciousness and not resulting from an external force (such as being pushed or hit). Exclusion criteria were self-reported: (i) presence of a macro-vascular condition (angina, stroke, peripheral vascular disease or diabetes) or a neuromuscular disease (multiple sclerosis, Alzheimer’s disease or Parkinson’s disease), (ii) use of a walking aid (cane or walker), (iii) ankle, knee or hip surgery ≤ 3 months, and/or (iv) pain of ≥ 8 on the Numeric Rating Scale (0—no pain at all, 10—worst pain imaginable).

### Outcome measurement tools

#### Kinematics

Lower limb kinematics were recorded using a 12-camera motion-capture system (Oqus-7, Qualisys AB, Gothenburg, Sweden; Fig. 1c) and a set of reflective markers on anatomical landmarks of the lower-extremities, pelvis and trunk^18^ (Fig. 1b) to capture 3-D body motion during walking at a frequency of 200 Hz. We placed reflective kinematic markers in the following locations: hallux, 1st and 5th metatarsal head, calcaneus and medial and lateral malleoli (directly on the footwear for the minimalist and supportive shoe conditions, and directly on the skin for barefoot condition), and head of the fibula, medial and lateral epicondyles, greater trochanter, anterior superior iliac spine, xyphoid process, jugular notch, 7th cervical vertebrae. Marker clusters were secured to the head, thorax (dorsally), sacrum, thighs and shanks using nylon/lycra bands. It is important to note that the foot length and width measurements, reported later in the results section, represent the external dimensions of the foot/shoe complex as defined by the marker placements, rather than the participant’s bare anatomical foot dimensions.

#### Kinetics

Three force platforms (Kistler Force Platform System 92-81B, Winterthur, Switzerland) embedded under the floor at the center of capture space (Fig. 1c) recorded ground reaction forces at 2000 Hz sampling rate and were synchronized to the marker-based system with a trigger.

#### Muscle activity

Surface electromyography (EMG) electrodes (Trigno Wireless; Delsys Inc., Boston, MA, United States) were placed on the dominant leg over the belly of the rectus femoris, vastus medialis, vastus lateralis, tibialis anterior, biceps femoris, semitendinosus, gastrocnemius medialis, and gastrocnemius lateralis according to the SENIAM (surface EMG for a non-invasive assessment of muscles) guidelines^19^. The dominant leg was defined as the leg that participants would choose to kick a ball. The EMG signals were collected at a sampling rate of 1000 Hz and amplified with a gain of 1000 (CMRR > 92 dB at 60 Hz, input impedance of 10 GW; 12 bits A/D converter). The skin was cleansed with alcohol swabs, shaved, and abraded with fine-grade sandpaper to reduce local impedance over the electrode implantation. EMG data were recorded using EMGworks software (Delsys Inc., Boston, MA, United States).

#### Procedures

After recording the participants’ age, sex, weight, and height, participants were fitted with the first footwear condition. The study coordinator (TC) fitted each participant with the shoes by palpating the participant’s hallux during standing to ensure that there was approximately 0.5–2 cm between the hallux and the shoe end. To ensure correct fitting, the participants were asked if they were comfortable in the shoe and if they felt it was appropriately fitted. To minimize participants’ bias, we did not inform the participants about the characteristics of the footwear or the study hypotheses. The participants were allowed to become accustomed to the new footwear condition by performing a 10-m walk ten times^20^. Prior to each trial, the cameras and force plates underwent calibration to guarantee consistent marker trajectories and reduce the impact of external noise. Participants were asked to wear standardized tight shorts. Participants were then seated, and surface EMG electrodes were placed at each of the selected muscles of the dominant leg. Then, kinematics markers were attached to specific bony landmarks.

Prior to walking trials, one trial was recorded in the standing position to define the segments’ coordinate systems. Following that, participants were instructed to perform walking trials across a 10-m walkway in two experimental conditions: (i) single task (straight walking), and (ii) dual task walking. Dual task walking involved simultaneous walking and counting backwards from a random number ranging from 50 to 200. All trials were conducted at the participant’s self-selected walking speed, and participants performed a minimum of six valid trials for each task, up to a maximum of ten trials. This range was implemented to ensure enough trials for analysis while preventing fatigue. A trial was repeated if it was invalid due to technical issues (e.g., marker occlusion) or a failure to correctly perform the task (e.g., not engaging in the counting-backwards task). During the walking trials, 3D body motion, GRFs and surface EMG were simultaneously captured. On completion of all walking trials in the first footwear condition, participants had a five-minute break, after which they were fitted with another type of footwear and repeated the assessments again, including the familiarization with new footwear.

### Data processing

#### Kinematics

Data collected using the motion capture system were exported as *.c3d files and then transformed into *.mat files, for further processing with biomechZoo^21^ and a custom MATLAB code (R2024a, The MathWorks Inc., Natick, MA, USA). 3-D trajectories of the markers were smoothed with a 4th order low-pass Butterworth filter (8 Hz cut-off frequency)^22^. Absolute thigh and shank angles were calculated as the motion of the segment relative to the laboratory coordinate system. The coordinate system of the segment was defined based on static trials performed with the participant in the orthostatic position. 3-D hip, knee and ankle angles were calculated as motion of the distant segment relative to the proximal segment via Euler angles (X = Frontal, Y = Sagittal and Z = Transverse).

#### Kinetics

**Fig. 2:**
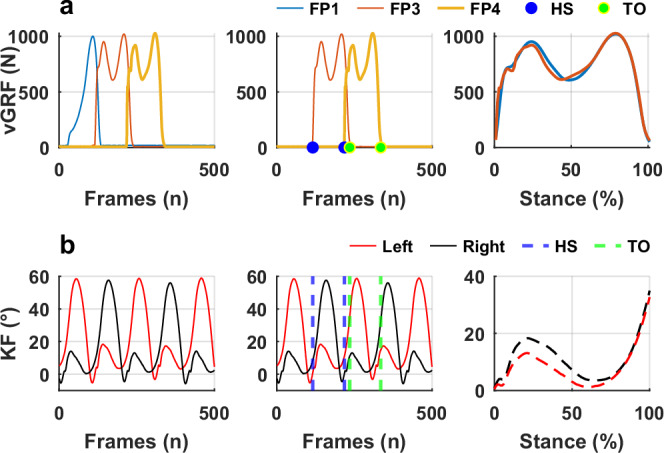
Data processing workflow for a random walking trial to depict how GRFs and joint kinematics were synchronized. **a** vertical GRF from three force plates (middle column: data from force plate 1 has been omitted in further processing). **b** knee flexion angles from both limbs with indices of heel strikes and toe offs to segment the angles based on stance phase. FP1—force plate 1; HS—heel strikes, TO—toe offs.

Ground reaction force data were collected using force plates and exported as *.c3d files, which were subsequently converted to *.mat format for further analysis in MATLAB. External joint moments at the ankle, knee, and hip were computed using a bottom-up inverse dynamics approach, applied separately for each limb and stance phase. The analysis was based on synchronized segment kinematics, ground reaction forces, and anthropometric parameters (Fig. 2)^22^. Heel contacts and toe-offs were determined using the vertical GRF with the threshold set at 50 N (Fig. 2, middle column). Segmental inertial properties, including mass, length, and moments of inertia, were estimated using established anthropometric models and assigned to the foot, shank, and thigh segments^23^. Joint center positions, segment center of mass (CoM) locations, CoM accelerations, segment angular velocities, and angular accelerations were all derived from motion capture data and interpolated to 101 points over the stance phase. 3D joint moments were resolved in the coordinate system of the proximal segment and normalized to body weight multiplied by height (BW × Height) to facilitate inter-participant comparison. Joint powers were then calculated as the scalar (dot) product of the joint moment and the corresponding angular velocity vector at each frame^24^. In line with convention, positive joint power values were defined as power generation, reflecting concentric muscle action and energy production, while negative joint power values were defined as power absorption, reflecting eccentric muscle action and energy dissipation.

#### Muscle activity

**Fig. 3:**
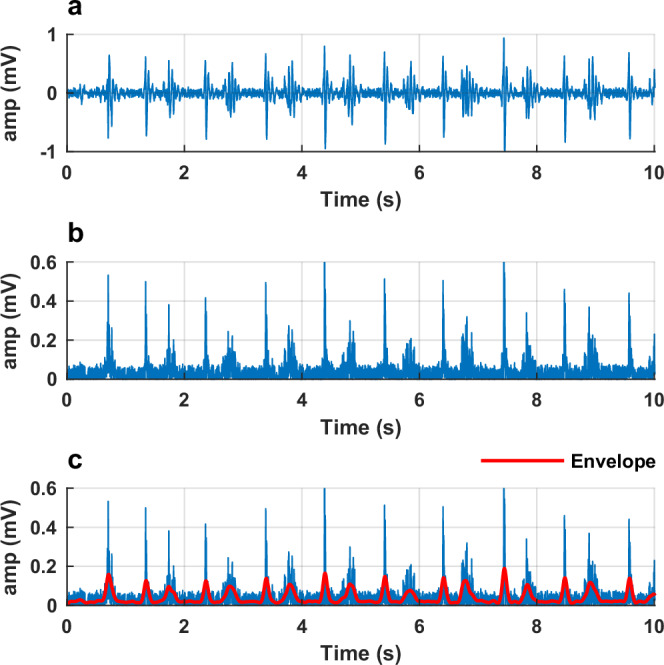
Example (gastrocnemius medialis) EMG data processing workflow from a random walking trial. **a** Raw data. **b** Filtered and rectified. **c** Linear envelope (red line).

EMG data were exported as *.csv files and then transformed into *.mat files. The raw signals (Fig. 3a) were full-wave rectified and passed through a zero-lag 2^nd^ order Butterworth low-pass filter with a band-pass filtered at 20–450 Hz (Fig. 3b). Then, we smoothed the signal with the 2^nd^ order Butterworth low-pass filter at 6 Hz to create a linear envelope of the signal (Fig. 3c)^25^. As recommended by the SENIAM guidelines, the amplitudes of the enveloped EMG signal were not normalized because this was a within-participant study design, i.e. we compared EMG amplitudes within a person and between randomly-assigned footwear conditions, without removing electrodes^26^.

#### Walking stability

We quantified the Margin of Stability (MoS)^27^ to express walking stability. The full description of how we did this and the effects of footwear on the MoS are presented in our previous study^7^. In summary, spatial position of the foot and pelvis markers were used as an input to quantify the MoS. The MoS is defined as the distance between the boundaries of the base of support (BoS) and the extrapolated position of the center of mass (XCoM) (Eq. 1)^27^. The position of the whole-body center of mass (CoM) was estimated as the three-dimensional centroid (average position) of the four pelvis markers (anterior and posterior superior iliac spines). This method provides a reliable estimate of the whole-body CoM trajectory for gait analysis, as the pelvis is a strong kinematic proxy that closely follows the path of the total body CoM^28,29^. The XCoM was calculated using Eq. (2).

1 $${MoS}={BoS}-{XCoM}$$

2 $${XCoM}=z+\left(\frac{x}{\sqrt{\frac{g}{l}}}\right)$$

Here, *z* is the CoM position, *x* is velocity of the CoM, *g* is the acceleration due to gravity (9.81 m/s²), and *l* is the average height of the COM over the whole walking trial^27^. Mean anterior-posterior and medio-lateral MoS were calculated at heel contact as the average of instantaneous MoS values for all the participant’s steps (left and right) in each footwear condition. To examine whether lower-limb biomechanics mediate the effect of minimalist footwear on walking stability in individuals with a history of falls, we focus here on the anterior–posterior (AP) MoS, which previously showed significant differences between footwear conditions^7^.

#### Motion capture and EMG synchronization

**Fig. 4:**
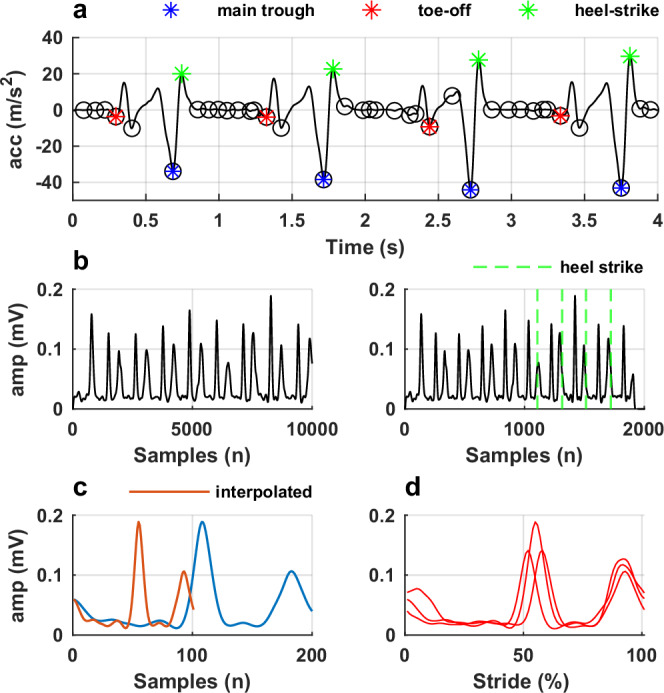
Processing pipeline for EMG temporal normalization. **a** Identification of gait events. **b** Resampling of the EMG signal and segmentation of the signal using time at heel-strike. **c** Interpolation of the EMG signal to 101 data points. **d** Segmentation of the EMG signal per all strides from a walking trial.

The motion capture system was configured to control start and stop of a recording from the force plates and the EMG system, by using a trigger. We used vertical acceleration of the reflective marker placed on the hallux to identify gait events i.e. heel-strike and toe-off, using *findpeaks* MATLAB function (Fig. 4a). We down sampled the processed EMG data to match sampling frequency of the motion capture, using the *resample* MATLAB function (Fig. 4b left). This enabled precise identification of gait events within the EMG signal (Fig. 4b right). This method was validated by visually inspecting the motion capture recording in the Qualisys Track Manager. To allow time-series comparisons, all data were normalized to 101 data points (Fig. 4c), using *interpft* function in MATLAB. We extracted data per stride (from one heel contact to the next heel contact of the same leg) (Fig. 4d). All data processing was carried out in MATLAB

### Statistics and reproducibility

First, to determine whether there is a difference in effect of footwear type on lower-extremity kinematics, kinetics and/or muscle activity, we performed a one-way repeated measures ANOVA using one-dimensional statistical parametric mapping (SPM)^30^. SPM enables continuous statistical analysis (across the whole signal waveform) without considering time points as independent. In the event of a significant effects, paired two-sided post-hoc tests with Bonferroni corrections were used to compare differences in outcomes by footwear type (barefoot vs supportive vs minimalist). SPM analysis was conducted in MATLAB, using the open source spm1d codes (https://www.spm1d.org).

Second, to validate the SPM results and examine whether cognitive load modifies the effect of footwear on lower-limb biomechanics, we performed linear mixed model analyses (LMM) with a footwear × walking condition interaction (single vs. dual task). The models employed a maximum likelihood estimation method with first-order autoregressive covariance type, and a random intercept was included to allow for variability across individuals (i.e., participant was treated as a random effect). Walking speed was included as a covariate in all models due to observed differences between footwear conditions (supplementary table 8). Additionally, for ankle kinematics and kinetics, the models were adjusted (i.e. included as covariates) for foot length and foot width as these differed depending on the footwear (Supplementary Table 8). As input to the models, we used mean angles at heel strike for joint kinematics or range values for joint kinetics and EMG amplitudes (max of the waveform—min of the waveform). LMM analyses were performed using SPSS software (IBM, Armonk, NY, USA; version 28.0).

**Fig. 5:**
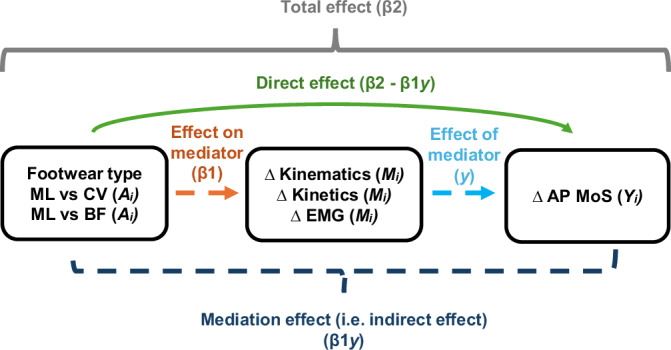
Visual representation of the modeling framework used to test the mediation effects. Mediation models provide estimates of the total effect, or “global” relationship between footwear type and gait stability (as expressed by the AP MoS) (β2; gray bracket), which is subdivided into estimates of the mediation effect β1γ. EMG—electromyography, BF—barefoot, CV—conventional supportive shoes, M—minimalist shoes, AP MoS—anterior-posterior Margin of Stability.

Third, we conducted mediation analyses to assess whether previously reported difference in effect of footwear on walking stability (MoS)^7^, is mediated by the difference in effect of footwear on lower-extremity biomechanics (Fig. 5). Mediation analysis is a statistical approach, which not only assesses whether an independent variable causes a change in a dependent variable, but also investigates how a third variable termed „mediator” contributes to this change. The indirect effect (i.e. mediation effect) was calculated based on the product of coefficient approach^31^. The significance of mediation effects was assessed with a 95% Confidence Intervals (CIs). To estimate CIs, we used a bias-corrected method with the percentile bootstrap estimation approach, which ran 5000 bootstrap iterations that were implemented. If the CIs included zero, we concluded that the indirect effect (i.e. mediation) was not significant. Conversely, the CIs that did not include zero suggested that there was a significant mediation. Positive values of the mediation effect indicate that the mediator transmits the effect of the independent variable (i.e., footwear condition) to the dependent variable (i.e. the MoS) in the same direction as the total effect. For example, if minimalist footwear increases rectus femoris activity and this increase is associated with an increase in the MoS, the mediation effect would be positive. In contrast, negative values of the mediation effect indicate that the mediator transmits the effect in the opposite direction, consistent with a suppressor effect. For instance, if minimalist footwear increases rectus femoris activity, but higher rectus femoris activity is associated with a decrease in the MoS, the mediation effect would be negative. Mediation analysis was implemented using MEMORE (Mediation and Moderation for Repeated Measures) macro for SPSS developed by Montoya and Hayes^32^. To further support the interpretation of mediation pathways, correlation analyses were performed and reported between within-subject differences in the mediator and outcome variables (e.g. ΔHip_Power vs. ΔMoS), providing an additional check on the directionality and strength of associations.

## RESULTS

### Participants’ characteristics

Thirty persons with a history of falls participated in the study. Biomechanical data (i.e. kinematics, GRFs and EMG) were not collected from two participants due to equipment malfunction during data acquisition. Additionally, data from three participants were excluded from EMG analyses because of excessive signal noise, and data from two participants were excluded from kinematic and kinetic analyses due to marker dropout and poor signal quality identified during post-processing. Further per-trial and per-participant inspection of EMG signals identified artefacts and low signal-to-noise ratio, leading to the exclusion of additional trials and the reporting of EMG data for 14–26 participants, depending on the muscle. The sample had a mean age of 68.1 ± 4.7 years, a mean height of 1.69 ± 0.08 m, a mean weight of 76.8 ± 13.9 kg, a mean BMI of 26.9 ± 3.8 kg/m², and 13 (50.0 %) were female. Participants walked more slowly when barefoot compared to walking in supportive shoes (mean difference (md)= 0.07 m/s). Foot length (difference between toe marker and heel marker) was lower when people were barefoot compared to when they wore supportive or minimalist shoes (md = 2.3 cm and md = 2.7 cm, respectively). Foot width (difference between marker on the 5^th^ metatarsal and 1^st^ metatarsal) was also lower when people were barefoot compared to when they wore supportive or minimalist shoes (md = 1.9 cm and md = 2.2 cm, respectively; Supplementary Table 8).

### Kinematics

#### Effects of footwear

**Fig. 6:**
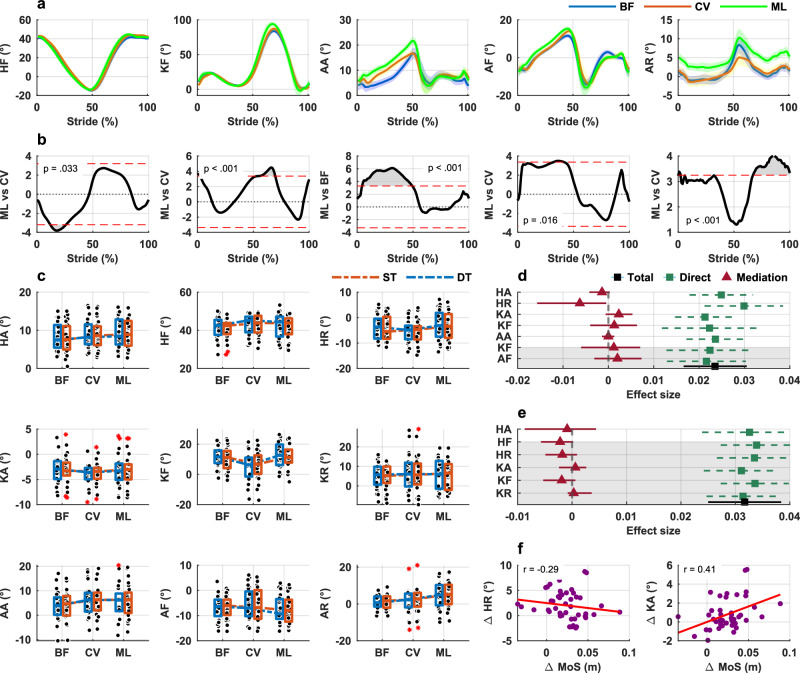
Joint kinematics and mediation analyses across footwear and cognitive task conditions. **a** Mean (SD) joint kinematics (angles) waveforms (averaged single and dual task) per footwear condition (*n* = 26). **b** Results of 1 D – SPM (corresponding to the waveforms presented in the row a), showing areas of significant differences between footwear comparisons (ML vs CV and ML vs BF for ankle abduction); t-critical is shown by the red dotted lines in each plot, statistical significance was assessed with two-sided paired-samples t-tests with Bonferroni correction. **c** Boxplots of joint kinematics values at heel strike stratified by footwear and walking condition, dashed line indicate any interaction between footwear and cognition; boxes represent the interquartile range (IQR), horizontal lines indicate the median, whiskers extend to 1.5× the IQR, individual points represent participant data, red asterisk denote outliers. (*n* = 26). **d** Results from the mediation analyses presented on the forest plots for the comparison between minimalist shoes and supportive shoes; total effect (with 95% Confidence Intervals) refers to the difference in the AP MoS between footwear conditions; significant mediation effect occurs if CIs do not cross 0; grayed out areas are for the range values of joint kinematics whereas white out areas are for the heel strike values of joint kinematics (*n* = 26). **e** Results from the mediation analyses for the comparison between minimalist shoes and barefoot; total effect (with 95% Confidence Intervals) refers to the difference in the AP MoS between footwear conditions (*n* = 26). **f** Scatterplots of the difference in MoS versus difference in hip rotation and knee abduction between minimalist shoes and supportive shoes (values at heel strike). % stride: 0—heel strike, 100—subsequent heel strike of the same limb, HA—hip abduction, HF—hip flexion, HR—hip rotation (same conventions applied to knee and ankle joints), BF—barefoot, CV—conventional supportive shoes, ML—minimalist shoes, ST—single task, DT—dual task, Δ—difference.

1-D SPM repeated measures ANOVA revealed statistically significant main effects of footwear on hip and knee flexion, and ankle in all three planes of movements (supplementary fig. 1). Post-hoc analyses (Fig. 6a, b) revealed that walking in minimalist shoes produced lower hip flexion than supportive shoes but greater hip flexion compared to walking barefoot. Furthermore, minimalist shoes led to greater knee flexion, and ankle dorsiflexion and external rotation compared to both barefoot and supportive shoes. Ankle abduction was lowest when walking barefoot, compared to both shod conditions. Mean (SD) waveforms per footwear type for all joints and planes of movement are presented in Supplementary Fig. 2. The mean ± SD number of strides per participant used for joint kinematics analysis was: 51.0 ± 14.7 for barefoot, 49.0 ± 15.5 for supportive shoes, and 54.2 ± 19.1 for minimalist shoes (supplementary table 1). Individual participants’ mean joint kinematics waveforms are presented in Supplementary Figs. 3–5.

#### Interaction effects

LMMs analysis did not show any statistically significant interaction between footwear and cognition (without and with additional cognitive load) for any of the joint kinematics outcomes independent whether we used joint angle values at heel strike (Fig. 6c, Supplementary Table 2), or range values as inputs to the models (Supplementary Table 3).

*Mediation effects*. Mediation analysis revealed that hip rotation at heel strike significantly mediated the difference in the MoS between minimalist and supportive shoes. (Fig. 6d). None of the joint kinematics outcomes mediated the difference in the MoS between minimalist shoes and barefoot (Fig. 6e).

### Kinetics

#### Effects of footwear

**Fig. 7:**
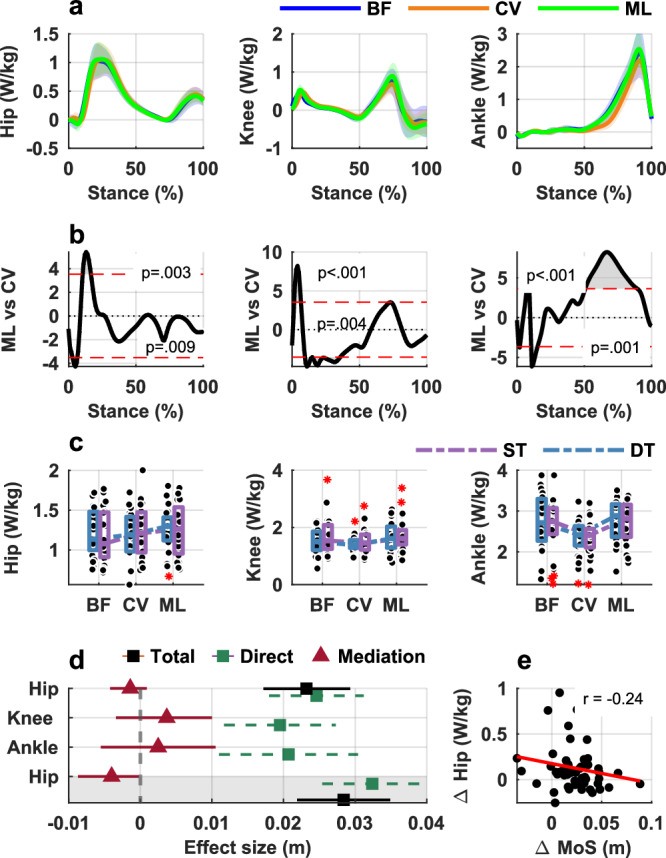
Joint kinetics and mediation analyses across footwear and cognitive task conditions. **a** Mean (SD) joint power waveforms (averaged single and dual task) per footwear condition (*n* = 26). **b** Results of 1 D – SPM (corresponding to the waveforms presented in row **a**) showing areas of significant differences between footwear comparisons; t-critical is shown by the red dotted lines in each plot, statistical significance was assessed with two-sided paired-samples t-tests with Bonferroni correction. **c** Boxplots of joint power range values stratified by footwear and walking condition (ST- single task, DT—dual task), dashed line indicate any interaction between footwear and cognition boxes represent the interquartile range (IQR), horizontal lines indicate the median, whiskers extend to 1.5× the IQR, individual points represent participant data, red asterisk denote outliers (*n* = 26). **d** Results from the mediation analyses presented on the forest plots for the comparison between minimalist shoes and supportive shoes, and, in the grayed out area, for the comparison between minimalist shoes and barefoot; total effect (with 95% Confidence Intervals) refers to the difference in the AP MoS between footwear conditions; significant mediation effect occurs if CIs do not cross 0 (*n* = 26). **e** Scatterplot of the difference in the MoS versus difference in hip power between minimalist shoes and barefoot (range values of joint powers were used). % stance: 0—heel strike, 100—toe off of the same limb, BF—barefoot, CV—conventional supportive shoes, ML—minimalist shoes, Δ—difference.

1-D SPM repeated measures ANOVA revealed statistically significant main effects of footwear on hip, knee, and ankle powers (Supplementary Fig. 6). Post-hoc analyses showed higher power generation (indicative of concentric muscle action) in the loading response when walking in minimalist shoes compared to walking in supportive shoes (Fig. 7a, b, left column). The analysis also showed lower knee power generation and higher knee power absorption (indicative of eccentric muscle action) in the loading response when walking in supportive shoes compared to walking in minimalist shoes (Fig. 7a, b, middle column). Finally, post-hoc analyses showed higher ankle power generation during the push-off phase when walking barefoot or in minimalist shoes compared to walking in supportive shoes (Fig. 7a, b, right column). Individual participants’ mean joint power waveforms are presented in Supplementary Fig. 7. The mean ± SD number of steps per participant used for joint kinetics analysis was: 20.1 ± 9.1 for barefoot, 18.1 ± 8.0 for supportive shoes, and 20.0 ± 9.0 for minimalist shoes (Supplementary Table 4).

#### Interaction effects

LMMs analysis did not show any statistically significant interaction between footwear and cognition for any of the joints independent whether range, peak positive or peak negative value of the outcome was used as an input to the models (Fig. 7c, Supplementary Table 5).

#### Mediation effects

None of the joint power outcomes mediated the difference in the MoS between minimalist shoes and supportive shoes. However, we observed that hip joint power mediated the difference in MoS between minimalist shoes and barefoot (Fig. 7d, grayed out area).

### Muscle activity

#### Effects of footwear

**Fig. 8:**
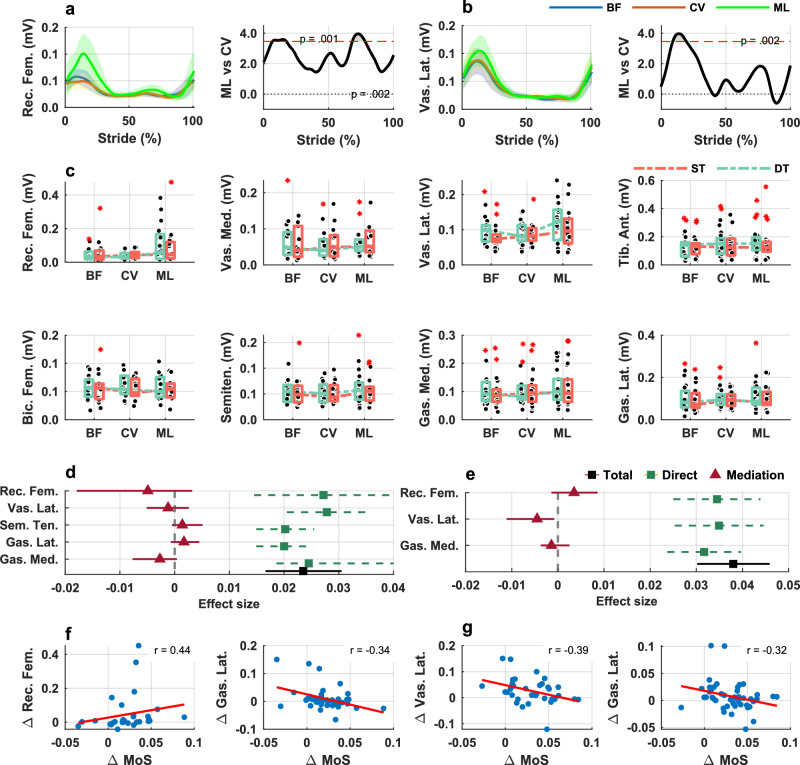
Muscle activity and mediation analyses across footwear and cognitive task conditions. **a**, **b** Mean (SD) EMG waveforms for rectus femoris and vastus lateralis (averaged single and dual task) per footwear condition (*n* = 1d4-26) with results of 1 D – SPM next to each plot, showing areas of significant differences between footwear comparisons; t-critical is shown by the red dotted lines in each plot, statistical significance was assessed with two-sided paired-samples t-tests with Bonferroni correction. **c** Boxplots of range values of EMG amplitudes stratified by footwear and walking condition; boxes represent the interquartile range (IQR), horizontal lines indicate the median, whiskers extend to 1.5× the IQR, individual points represent participant data, red asterisk denote outliers (*n* = 14–26). **d** Rfesults from the mediation analyses presented on the forest plots for the comparison between minimalist shoes and supportive shoes; total effect (with 95% Confidence Intervals) refers to the difference in the AP MoS between footwear conditions; significant mediation effect occurs if CIs do not cross 0 (*n* = 14-26). **e** Results from the mediation analyses for the comparison between minimalist shoes and barefoot (*n* = 14-26). **f** Scatterplots of the difference in the MoS versus difference in amplitude of rectus femoris and gastrocnemius lateralis between minimalist shoes and supportive shoes. **g** Scatterplots of the difference in the MoS versus difference in amplitude of vastus lateralis and gastrocnemius lateralis between minimalist shoes and barefoot. % stride: 0—heel strike, 100—subsequent heel strike of the same limb, mV—miliVolts, BF—barefoot, CV—conventional supportive shoes, ML—minimalist shoes, ST—single task, DT—dual task, Δ—difference; Participant numbers (*n*) per muscle: Rec. Fem: BF = 18, CV = 17, ML = 17; Vas. Lat.: BF = 20, CV = 23, ML = 18; Vas. Med: BF = 17, CV = 16, ML = 14; Tib. Ant.: BF = 24, CV = 26, ML = 24; Bic. Fem: BF = 15, CV = 14, ML = 14; Semiten.: BF = 22, CV = 23, ML = 24; Gas. Lat.: BF = 26, CV = 25, ML = 24; Gas. Med: BF = 23, CV = 24, ML = 23.

1-D SPM repeated measures ANOVA revealed statistically significant main effects of footwear on EMG amplitudes of rectus femoris and vastus lateralis (Supplementary Fig. 8). Post-hoc analyses showed that walking in minimalist shoes resulted in higher activity of rectus femoris (Fig. 8a) and higher activity of vastus lateralis (Fig. 8b) compared to walking in supportive shoes. The mean ± SD number of strides per participant used for the EMG analysis was: 25.6 ± 7.7 for barefoot, 24.2 ± 5.7 for supportive shoes, and 25.4 ± 5.1 for minimalist shoes (Supplementary Table 6). Mean (SD) EMG waveforms for all muscles are presented in Supplementary Fig. 9. Individual participants’ mean EMG waveforms are presented in Supplementary Fig. 10–12.

#### Interaction effects

LMMs analysis, using range values of EMG amplitudes as input, did not show any statistically significant interaction between footwear and cognition (Fig. 8c, Supplementary Table 7).

#### Mediation effects

None of the EMG outcomes mediated the difference in the MoS between minimalist shoes and supportive shoes (Fig. 8d). However, we observed that activity of vastus lateralis mediated the difference in MoS between minimalist shoes and barefoot (Fig. 8e).

## Discussion

To our knowledge, this is the first study that investigated the effects of footwear type on lower extremity biomechanics during walking in persons with a history of falls. Walking in minimalist shoes compared to supportive shoes resulted in notable differences in joint kinematics, kinetics, and muscle activity.

First, in terms of joint kinematics, we observed lower hip flexion during the stance phase, greater knee flexion during the swing phase, greater ankle flexion during the stance phase, and increased ankle external rotation during swing phase when participants walked in minimalist shoes compared to when walking in supportive shoes. The reduced hip flexion during stance may indicate a more neutral alignment of the lower extremity in minimalist shoes, possibly due to the reduced heel-to-toe drop and lack of cushioning. This could promote a more natural gait pattern, requiring less compensatory hip movement to stabilize the CoM. Our findings also showed a significant increase in ankle dorsiflexion in minimalist shoes during the late stance phase which primarily reflects passive motion of the shank rolling over the foot, rather than active contraction of the dorsiflexor muscles. This increased dorsiflexion extends the range of motion through which the plantar flexors can generate force, lengthening the ‘propulsive lever arm’ and contributing to the higher ankle power generation observed during push-off. Therefore, the primary kinematic mechanism underpinning the improved propulsion in minimalist shoes is not an alteration at initial contact, but rather the enabling of a greater and potentially more effective ankle rocker function in terminal stance. This might be a direct consequence of the minimalist shoe’s reduced heel-to-toe drop and minimal cushioning, which permit a more extensive use of the ankle’s sagittal plane range of motion. We also observed greater knee flexion during the swing phase in minimalist shoes which may represent a compensatory adjustment to ensure adequate foot clearance in the absence of additional structural support from the footwear. While this is an effective strategy to prevent tripping, it is worth noting that foot clearance controlled primarily through the knee joint can exhibit higher variability in toe clearance compared to a more ankle-centric strategy—a factor associated with increased tripping risk^33^. Therefore, this adaptation, although functionally necessary, may represent a different control mechanism that might place greater demands on proximal neuromuscular coordination. As such one must consider both the potential benefits and drawbacks of such adaptations when interpreting the stability-related effects of minimalist footwear.

Second, footwear significantly influenced lower-limb joint power during walking in persons with a history of falls. Specifically, walking in minimalist shoes—compared to supportive shoes—resulted in greater power generation at the hip during loading response and at the ankle during push-off. These results suggest that minimalist footwear supports a more dynamic and responsive gait pattern, characterized by energy generation at key phases of the gait cycle. Greater hip power generation during the loading response indicates more effective control of forward progression and stabilization of the upper body at foot contact. This is particularly important in older adults, who often exhibit diminished hip extensor function, contributing to reduced ability to recover from external perturbations. Insufficient hip power has been associated with impaired stability control, shortened step length, and decreased walking speed—established risk factors for falls in older adults^34^. At the ankle, the increased power generation during push-off in minimalist shoes reflects a greater capacity for propulsion from the plantar flexors. This is consistent with the known role of the plantar flexors in providing forward thrust and maintaining momentum during the late stance phase^35^. While an optimal stretch-shortening cycle would utilize energy absorbed in early stance, the increased generation we observed indicates a strategy reliant on significant concentric muscle action. Previous research has shown that reduced ankle push-off power in older adults is linked to slower gait, impaired obstacle negotiation, and compromised ability to respond to sudden loss of stability^36^. For older adults with a history of falls, who often exhibit age-related declines in ankle power, this ability to generate strong push-off is important for maintaining walking speed, clearing obstacles, and performing effective compensatory steps to avoid a fall. In contrast, the greater power absorption and lower power generation at the knee observed in supportive footwear may suggest a more passive gait strategy, potentially due to the mechanical constraints of the shoe. Although this may reduce muscular effort, it can also limit the neuromechanical responsiveness of the lower limb. Effective eccentric control at the knee is critical not only for maintaining stability during single-limb support and recovery from perturbations but also for protecting the joint by absorbing impact loads and reducing stress on the articular cartilage^37^. Impairments in this control are said to be related to mediolateral instability, reduce the effectiveness of protective stepping responses, and contribute to degenerative changes in the knee joint^38,39^—factors that may collectively elevate fall and injury risk.

Third, we observed higher activity of the rectus femoris (RF) and vastus lateralis (VL) during the stance phase and higher activity of the RF during the swing phase when participants walked in minimalist shoes compared to when walking in supportive shoes. This neuromuscular adaptation is directly supported by the concurrent kinetic findings. As shown in Fig. 8a, b, minimalist shoes were associated with increased knee power generation at heel strike and lower power absorption during the loading response. This pattern suggests that the elevated quadriceps activity facilitates an active, power-generating strategy to control the limb. Rather than a more passive, dampening response seen in supportive shoes, the neuromuscular system in minimalist footwear appears to use concentric-based quadriceps action to swiftly stabilize the knee and control the CoM during the critical weight-acceptance phase. The biarticular RF is critical for gait, because it helps with hip flexion and knee extension during the swing phase to achieve proper step length and prepare the foot for initial contact. This ensures efficient forward progression into the stance phase^40^. The VL primarily stabilizes and extends the knee during stance, eccentrically controlling the loading response and producing concentric force for body weight support during mid-stance. Older adults demonstrate deficits in eccentric knee extensor control during single limb support after external perturbations, which contributes to greater postural sway during balance recovery^41^. Furthermore, lower thickness and cross-sectional area of RF have recently been shown to predict falls in geriatric outpatients^42^. This highlights the importance of the knee extensor for stabilizing the CoM during stability responses to external perturbations, eccentric control, and to provide postural stability in the single leg support phase for the successful protective stepping^43^. Therefore, the observed increase in RF and VL activity in minimalist shoes likely represents a beneficial adaptive mechanism. It enhances proximal joint control and stabilizes the CoM under the heightened sensorimotor demands imposed by reduced external footwear support, potentially leading to more effective management of gait perturbations.

Regarding the second research question, we found that the effects of footwear type on lower-limb joint kinematics, joint powers, and muscle activity were not significantly altered by the addition of a cognitive task. That is, minimalist footwear influenced gait biomechanics in a consistent manner across both single-task and dual-task walking conditions. This suggests that the neuromechanical adaptations induced by footwear are relatively robust and not strongly modulated by the moderate level of cognitive load introduced in this study. We initially expected that cognitive loading might amplify the effects of footwear, given the well-established vulnerability of postural control systems to dual-task interference in older adults. Stability during walking depends on the timely integration of vestibular, visual, and proprioceptive inputs, all of which can deteriorate with age. Moreover, cognitive-motor interference has been shown to exaggerate gait instability and impair postural control, particularly in populations with mild cognitive impairment or frailty^44^. In such contexts, the central nervous system may rely more heavily on peripheral sensory input—including plantar cutaneous feedback—for stability, especially when attentional resources are impacted. Minimalist footwear, which enhances plantar sensory feedback, was thought to play a compensatory role under cognitive load by increasing reliance on somatosensory input from the feet. However, our findings do not support this interaction effect, which might be due to several plausible explanations. First, the cognitive challenge (serial subtraction) may not have been sufficiently demanding or complex to force a shift in motor strategy. A more difficult task (e.g., category switching, auditory stroop) that places higher demands on executive function might be necessary to elicit this interaction. Second, our participants, while fall-prone, were relatively young and cognitively intact (mean age ≈ 68 years). In a cohort with more pronounced executive dysfunction or frailty, the reliance on enhanced plantar feedback from minimalist shoes under cognitive load might become more critical. These considerations raise questions about ecological validity. The contextual complexity and unpredictability that exacerbate cognitive-motor interference in daily life were probably reduced in our controlled laboratory environment. It is unclear whether these gait adaptations would be maintained or even magnified during more ecologically challenging scenarios, such as walking in a crowded space, negotiating cluttered environments, or reacting to an unexpected perturbation. Further research using more demanding cognitive tasks, ecologically valid environments, or populations with cognitive impairment is needed to determine whether footwear effects interact with cognitive load in real-world contexts.

Finally, we observed distinct and condition-specific mediation effects, suggesting that multiple neuromechanical mechanisms contribute to how minimalist shoes influence gait stability in persons with a history of falls. Specifically, we found that hip external rotation at heel strike significantly mediated the difference in walking stability between minimalist and supportive shoes. This indicates that alterations in limb orientation at initial contact partly account for how minimalist footwear modifies dynamic stability relative to supportive designs. Conversely, no significant mediating effects were found among joint kinetic or muscle activity variables for this comparison, suggesting that kinematic strategies—rather than changes in force production or neuromuscular effort—are the dominant mechanism in this context. When comparing minimalist shoes to barefoot walking, two distinct mediators emerged: hip joint power and vastus lateralis muscle activity. In both cases, an increase in these variables was associated with a reduction in the MoS. Specifically, in minimalist shoes, an increase in hip power and vastus lateralis activity was associated with a reduction in the anterior-boundary MoS. This reflects a more anteriorly positioned momentum at heel strike, which is indicative of a more dynamically stable configuration against backward balance loss. These differences may reflect small changes in mechanical interaction with the ground—such as sole compliance or friction—that influence how sensory information is integrated and how proximal joints contribute to maintaining dynamic stability. This pattern suggests that minimalist shoes may facilitate a more coordinated distribution of mechanical effort across the lower limb, supporting stable forward progression. Taken together, these findings underscore the central role of proximal joint function—particularly at the hip—in mediating footwear-related changes in dynamic stability. Hip rotation at heel strike influences lower limb alignment and foot orientation when the base of support is established, directly affecting forward momentum. Similarly, increases in hip joint power and quadriceps (vastus lateralis) activity may reflect more efficient movement patterns or improved postural control, potentially minimizing destabilizing forces during early stance. These biomechanical changes are highly relevant to the AP MoS, which quantifies the difference between the xCoM and the anterior boundary of the base of support, defined by foot placement at heel strike. Variables that influence xCoM trajectory—such as CoM velocity, trunk control, and foot placement timing—are therefore mechanistically linked to the MoS. While our study was not designed to directly evaluate fall incidence, these findings identify proximal joint mechanics as plausible biomechanical pathways through which footwear may influence walking stability. By identifying specific neuromechanical mediators, our results provide an important foundation for understanding the biomechanical mechanisms through which footwear influences gait, which is a necessary precursor for designing future clinical trials. Nevertheless, future longitudinal studies will be required to confirm whether such mechanisms translate into real-world fall reduction.

In contrast, we did not observe significant mediation through other lower-limb kinematic or muscle activity variables, including those at the ankle or knee. There are several possible explanations. First, the mechanical contributions of the ankle and knee may influence other aspects of gait performance (e.g., propulsion, energy efficiency) more than dynamic stability as measured by the MoS AP. Second, while muscle activation patterns changed in response to footwear, their relationship to CoM control and foot placement may be indirect or modulated through higher order neuromotor coordination not captured in this analysis. This absence of broader mediation effects may also be due, in part, to the limited sample size. Based on our data, a sample of approximately 200 participants would be required to achieve 80% power to detect small-to-moderate indirect effects (approximately 0.13) in a within-subject design^45^. While the current sample size may have limited the detection of more subtle mediating effects, it was sufficient to reveal key biomechanical factors—particularly at the hip—highlighting robust, functionally meaningful adaptations in walking stability with minimalist footwear. Despite these limitations, these findings improve our understanding of how footwear influences neuromechanical control of gait stability in older adults and thus support the development of interventions that target hip mechanics—either through training or assistive technologies—and the need to consider proximal joint dynamics when evaluating fall prevention strategies.

The fundamental difference between minimalist and supportive footwear lies in the degree of plantar cutaneous stimulation and the mechanical freedom afforded to the foot. Supportive shoes, with their cushioning and motion control features, likely dampen plantar sensory feedback and restrict natural foot motion, leading the sensorimotor system to adopt a more passive, constrained gait strategy. This is evidenced by our findings of lower ankle power generation and an absorption-dominant strategy at the knee. In contrast, the thin, flexible soles of minimalist shoes may enhance the transmission of tactile and pressure cues from the plantar surface to the central nervous system. This richer afferent signal appears to facilitate a shift in motor control strategy towards a more active and dynamic gait. The observed increases in ankle and hip power generation, greater ankle mobility, and elevated muscle activity reflect this shift. The neuromuscular system, receiving more precise and timely information about foot-ground interactions, is better able to execute precise, power-generating movements for propulsion and stability. The identified mediation of stability adaptations through proximal joint mechanics further suggests that enhanced foot-level feedback drives adaptive changes in whole-limb coordination. Therefore, the biomechanical differences observed are not merely isolated changes but may be manifestations of a fundamentally different, more responsive sensorimotor control strategy induced by minimalist footwear. While the current study was not designed to directly measure plantar sensitivity, this proposed mechanism, while supported by the findings, remains inferential. Future research should aim to directly quantify this relationship. For instance, measuring foot tactile sensitivity using Semmes-Weinstein monofilaments and using this data as a moderator in biomechanical analyses could elucidate whether individual differences in sensory capacity predict the magnitude of footwear-induced gait adaptations. This would move beyond speculation and provide direct evidence for the role of cutaneous afference in mediating the effects of footwear on gait control.

This study has several limitations. First, although participants were selected based on a history of falls, the sample was relatively young (mean age <70 years), which may limit generalizability to older and more frail populations who are at higher risk of falls and may exhibit greater gait instability or cognitive-motor interference. Second, the absence of a healthy age-matched control group prevents direct comparisons to baseline gait biomechanics in individuals without a history of falls. As a result, it is unclear whether the observed biomechanical adaptations represent deviations from, or normalization toward, typical gait patterns. Third, all assessments were conducted in a controlled laboratory environment, which does not fully reflect real-world walking, which often involves variable terrain, environmental distractions, and unexpected perturbations. Moreover, only one model of supportive footwear was tested. Although previous work suggests that minimalist shoes produce consistent effects across models in terms of center of pressure metrics^8^, we cannot exclude the possibility that different supportive shoe designs—e.g., with variations in sole stiffness, rocker profiles, or heel elevation—might yield different biomechanical outcomes. Furthermore, our finding of larger AP MoS in minimalist footwear should be interpreted with caution regarding its relationship to fall risk. As recently elaborated by Curtze et al.^46^ and Watson et al.^47^, the MoS is an instantaneous measure of mechanical stability and should not be equated with global gait stability or general fall risk. In fact, larger MoS values are often observed in populations with an increased risk of falls as a compensatory strategy for underlying neuromotor deficits^46^. Therefore, we interpret the larger MoS when walking in minimalist shoes in our fall-prone participants not as a direct enhancement of stability against backward falls, but as a reflection of an altered gait strategy. This strategy, characterized by a more posteriorly directed momentum at initial contact, may represent a cautious adaptation to the enhanced sensory feedback and reduced external support of minimalist footwear. This interpretation aligns with the other observed biomechanical changes, such as increased proximal muscle activity, suggesting a shift towards a more actively controlled gait pattern. Finally, a potential limitation of this single-session study is that the observed biomechanical adaptations could be interpreted as transient responses to the unfamiliarity of the minimalist footwear, rather than a stable neuromotor strategy. However, two aspects of our study suggest the effects are specific to the footwear’s properties. First, participants completed an acclimatization period of approximately 100 meters of walking in each condition to mitigate initial novelty effects. Second, we observed significant differences in the MoS between the minimalist and supportive shoes as well as between minimalist shoes and barefoot, but not between the barefoot and supportive shoes. If unfamiliarity alone were the primary driver, one would expect the most unfamiliar condition (barefoot) to also differ significantly from the supportive shoes, which was not the case. This pattern suggests that the adaptations are a specific response to the unique sensorimotor context of minimalist footwear. Nonetheless, longitudinal studies are required to confirm whether these adaptations persist with long-term use and represent a durable change in gait control.

## Conclusions

This study demonstrates that minimalist footwear modifies lower-limb biomechanics in individuals with a history of falls, by increasing joint mobility, hip and ankle power generation, and activation of key stabilizing muscles such as the rectus femoris and vastus lateralis. These adaptations reflect a shift toward a more active and responsive gait strategy. Importantly, mediation analysis identified that changes in hip kinematics, hip joint power, and quadriceps activity are plausible mediators that may partially explain the association between minimalist footwear and dynamic stability. These effects were consistent across cognitive load conditions, suggesting robust neuromechanical responses to footwear. Taken together, these associative results provide new, hypothesis-generating insight into potential mechanisms through which footwear influences gait stability and justify evaluating minimalist shoes in future fall-prevention intervention trials.

## Supplementary information

Supplementary Material

Description of Additional Supplementary Files

Supplementary Data 1

Supplementary Data 2

Supplementary Data 3

Supplementary Data 4

Supplementary Data 5

Supplementary Data 6

## Data Availability

All source data are available in the Supplementary Data. The source data for Fig. 6a, b are provided in Supplementary Data 1, which include individual participants’ mean (average left and right limb) hip, knee and ankle 3D joint angle waveforms (from heel strike to subsequent heel strike of the same limb) stratified by footwear and walking condition (single task and dual-task). The source data for Fig. 6c–f are provided in Supplementary Data 2, which include (i) anterior–posterior MoS values at heel strike and (ii) hip, knee, and ankle joint angle ranges and heel-strike angles for each participant, stratified by footwear and walking condition (single task and dual-task). The source data for Fig. 7a, b are provided in Supplementary Data 3, which include individual participants’ mean (average left and right limb) hip, knee and ankle joint power waveforms (from heel strike to toe off of the same limb) stratified by footwear and walking condition (single task and dual-task). The source data for Fig. 7c–e are provided in Supplementary Data 4, which include (i) anterior–posterior MoS values at heel strike and (ii) hip, knee, and ankle joint power range values for each participant, stratified by footwear and walking condition. The source data for Fig. 8a, b are provided in Supplementary Data 5, which include individual participants’ mean EMG waveforms (from heel strike to subsequent heel strike of the same limb) for each muscle stratified by footwear and walking condition (single task and dual-task). The source data for Fig. 8c–g are provided in Supplementary Data 6, which include (i) anterior–posterior MoS values at heel strike and (ii) EMG range values for each muscle and participant, stratified by footwear and walking condition (single task and dual-task).

## Code availability

All codes underpinning the results of this study are available in figshare with the identifier DOI: 10.6084/m9.figshare.29588918^48^.

## References

[1] Strzalkowski, N. D. J., Peters, R. M., Inglis, J. T. & Bent, L. R. Cutaneous afferent innervation of the human foot sole: what can we learn from single-unit recordings? *J. Neurophysiol.* **120**, 1233–1246 (2018). 29873612 10.1152/jn.00848.2017 PMC6171067

[2] Fallon, J. B., Bent, L. R., McNulty, P. A. & Macefield, V. G. Evidence for strong synaptic coupling between single tactile afferents from the sole of the foot and motoneurons supplying leg muscles. *J. Neurophysiol.* **94**, 3795–3804 (2005). 16079197 10.1152/jn.00359.2005

[3] Rubenstein, L. Z. Falls in older people: epidemiology, risk factors and strategies for prevention. *Age Ageing* **35**, 2–ii37-ii41 (2006). 10.1093/ageing/afl084 16926202

[4] McKeon, P. O., Hertel, J., Bramble, D. & Davis, I. The foot core system: a new paradigm for understanding intrinsic foot muscle function. *Br. J. Sports Med.* **49**, 290 (2015). 24659509 10.1136/bjsports-2013-092690

[5] Davis, A., Haines, T. & Williams, C. Do footwear styles cause falls or increase falls risk in healthy older adults? A systematic review. *Footwear Sci.* **11**, 13–23 (2019).

[6] Menz, H. B., Morris, M. E. & Lord, S. R. Footwear characteristics and risk of indoor and outdoor falls in older people. *Gerontology* **52**, 174–180 (2006). 16645298 10.1159/000091827

[7] Cudejko, T., Gardiner, J., Akpan, A. & D’Août, K. Minimal shoes improve stability and mobility in persons with a history of falls. *Sci. Rep.* **10**, 21755 (2020). 33303964 10.1038/s41598-020-78862-6 PMC7730448

[8] Cudejko, T., Gardiner, J., Akpan, A. & D’Août, K. Minimal footwear improves stability and physical function in middle-aged and older people compared to conventional shoes. *Clin. Biomech.* **71**, 139–145 (2020). 10.1016/j.clinbiomech.2019.11.005 31739197

[9] Petersen, E., Zech, A. & Hamacher, D. Walking barefoot vs. with minimalist footwear – influence on gait in younger and older adults. *BMC Geriatrics* **20**, 88 (2020). 32131748 10.1186/s12877-020-1486-3 PMC7057536

[10] Ren, X. et al. Barefoot walking is more stable in the gait of balance recovery in older adults. *BMC Geriatrics* **22**, 904 (2022). 36434546 10.1186/s12877-022-03628-w PMC9700923

[11] Esculier, J.-F., Dubois, B., Dionne, C. E., Leblond, J. & Roy, J.-S. A consensus definition and rating scale for minimalist shoes. *J. Foot Ankle Res.* **8**, 42 (2015). 26300981 10.1186/s13047-015-0094-5 PMC4543477

[12] Willems, C., Curtis, R., Pataky, T. & D’Août, K. Plantar pressures in three types of indigenous footwear, commercial minimal shoes, and conventional Western shoes, compared to barefoot walking. *Footwear Sci.* **13**, 1–17 (2020).

[13] Franklin, S., Li, F.-X. & Grey, M. J. Modifications in lower leg muscle activation when walking barefoot or in minimalist shoes across different age-groups. *Gait Posture* **60**, 1–5 (2018). 29121509 10.1016/j.gaitpost.2017.10.027

[14] Hannigan, J. J. & Pollard, C. D. Comparing walking biomechanics of older females in maximal, minimal, and traditional shoes. *Gait Posture* **83**, 245–249 (2021). 33197860 10.1016/j.gaitpost.2020.10.030

[15] Franklin, S., Grey, M. J., Heneghan, N., Bowen, L. & Li, F.-X. Barefoot vs common footwear: a systematic review of the kinematic, kinetic and muscle activity differences during walking. *Gait Posture* **42**, 230–239 (2015). 26220400 10.1016/j.gaitpost.2015.05.019

[16] Hausdorff, J. M., Schweiger, A., Herman, T., Yogev-Seligmann, G. & Giladi, N. Dual-task decrements in gait: contributing factors among healthy older adults. * J. Gerontology: Ser. A* **63**, 1335–1343 (2008). 10.1093/gerona/63.12.1335 PMC3181497 19126846

[17] von Elm, E. et al. The Strengthening the Reporting of Observational Studies in Epidemiology (STROBE) statement: guidelines for reporting observational studies. *J. Clin. Epidemiol.* **61**, 344–349 (2008). 18313558 10.1016/j.jclinepi.2007.11.008

[18] Cappozzo, A., Catani, F., Croce, U. D. & Leardini, A. Position and orientation in space of bones during movement: anatomical frame definition and determination. *Clin. Biomech. (Bristol, Avon)* **10**, 171–178 (1995). 10.1016/0268-0033(95)91394-t 11415549

[19] Hermens, H. J., Freriks, B., Disselhorst-Klug, C. & Rau, G. Development of recommendations for SEMG sensors and sensor placement procedures. *J. Electromyogr. Kinesiol.* **10**, 361–374 (2000). 11018445 10.1016/s1050-6411(00)00027-4

[20] Melvin, J. M. A., Preece, S., Nester, C. J. & Howard, D. An investigation into plantar pressure measurement protocols for footwear research. *Gait Posture* **40**, 682–687 (2014). 25161007 10.1016/j.gaitpost.2014.07.026

[21] Dixon, P. C., Loh, J. J., Michaud-Paquette, Y. & Pearsall, D. J. biomechZoo: An open-source toolbox for the processing, analysis, and visualization of biomechanical movement data. *Comput. Methods Prog. Biomed.* **140**, 1–10 (2017). 10.1016/j.cmpb.2016.11.007 28254065

[22] Winter, D. A. Human balance and posture control during standing and walking. *Gait Posture* **3**, 193–214 (1995).

[23] Winter, D. A. in *Biomechanics and Motor Control of Human Movement* 82–106 (2009).

[24] Farris, D. J. & Sawicki, G. S. The mechanics and energetics of human walking and running: a joint level perspective. *J. R. Soc. Interface* **9**, 110–118 (2011). 21613286 10.1098/rsif.2011.0182 PMC3223624

[25] Merletti, R. & Cerone, G. L. Tutorial. Surface EMG detection, conditioning and pre-processing: Best practices. *J. Electromyogr. Kinesiol.* **54**, 102440 (2020). 32763743 10.1016/j.jelekin.2020.102440

[26] Besomi, M. et al. Consensus for experimental design in electromyography (CEDE) project: amplitude normalization matrix. *J. Electromyogr. Kinesiol.* **53**, 102438 (2020). 32569878 10.1016/j.jelekin.2020.102438

[27] Hof, A. L., Gazendam, M. G. & Sinke, W. E. The condition for dynamic stability. *J. Biomech.* **38**, 1–8 (2005). 15519333 10.1016/j.jbiomech.2004.03.025

[28] Eames, M. H. A., Cosgrove, A. & Baker, R. Comparing methods of estimating the total body centre of mass in three-dimensions in normal and pathological gaits. *Hum. Mov. Sci.* **18**, 637–646 (1999).

[29] Wada, O., Tateuchi, H. & Ichihashi, N. The correlation between movement of the center of mass and the kinematics of the spine, pelvis, and hip joints during body rotation. *Gait Posture* **39**, 60–64 (2014). 23810089 10.1016/j.gaitpost.2013.05.030

[30] Pataky, T. C. Generalized n-dimensional biomechanical field analysis using statistical parametric mapping. *J. Biomech.* **43**, 1976–1982 (2010). 20434726 10.1016/j.jbiomech.2010.03.008

[31] MacKinnon, D. P., Lockwood, C. M., Hoffman, J. M., West, S. G. & Sheets, V. A comparison of methods to test mediation and other intervening variable effects. *Psychol. Methods* **7**, 83–104 (2002). 11928892 10.1037/1082-989x.7.1.83 PMC2819363

[32] Montoya, A. K. & Hayes, A. F. Two-condition within-participant statistical mediation analysis: a path-analytic framework. *Psychol. Methods* **22**, 6–27 (2017). 27362267 10.1037/met0000086

[33] Barrett, R. S., Mills, P. M. & Begg, R. K. A systematic review of the effect of ageing and falls history on minimum foot clearance characteristics during level walking. *Gait Posture* **32**, 429–435 (2010). 20692163 10.1016/j.gaitpost.2010.07.010

[34] Smith, T. O. et al. Clinical and biomechanical factors associated with falls and rheumatoid arthritis: baseline cohort with longitudinal nested case–control study. *Rheumatology* **61**, 679–687 (2022). 33905483 10.1093/rheumatology/keab388 PMC8824410

[35] Sutherland, D. H., Cooper, L. & Daniel, D. The role of the ankle plantar flexors in normal walking. *JBJS* **62** (1980). 7364808

[36] Franz, J. R. The age-associated reduction in propulsive power generation in walking. *Exercise Sport Sci. Rev.* **44** (2016). 10.1249/JES.0000000000000086 PMC9382873 27433977

[37] Vincent, K. R., Conrad, B. P., Fregly, B. J. & Vincent, H. K. The pathophysiology of osteoarthritis: a mechanical perspective on the knee joint. *PM R.* **4**, S3–S9 (2012). 22632700 10.1016/j.pmrj.2012.01.020 PMC3635670

[38] Peitola, J. P. J., Esrafilian, A., Simonsen, M. B., Andersen, M. S. & Korhonen, R. K. Reduced muscle strength can alter the impact of gait modifications on knee cartilage mechanics. *J. Orthop. Res.* **43**, 1566–1580 (2025). 40576011 10.1002/jor.70007 PMC12329646

[39] Lee, P.-A. et al. Compromised balance control in older people with bilateral medial knee osteoarthritis during level walking. *Sci. Rep.* **11**, 3742 (2021). 33580161 10.1038/s41598-021-83233-w PMC7881198

[40] Frigo, C. A., Wyss, C. & Brunner, R. The effects of the rectus femoris muscle on knee and foot kinematics during the swing phase of normal walking. *Applied Sciences* **10** (2020).

[41] Jeon, W., Whitall, J., Alissa, N. & Westlake, K. Age-related differences in stepping stability following a sudden gait perturbation are associated with lower limb eccentric control of the perturbed limb. *Exp. Gerontol.* **167**, 111917 (2022). 35963451 10.1016/j.exger.2022.111917 PMC13085867

[42] Güner, M. et al. The role of ultrasonographically measured rectus femoris muscle on falls in community-dwelling older adults: a single-center study. *Eur. Geriatr. Med.* **14**, 1065–1073 (2023). 37353629 10.1007/s41999-023-00823-9

[43] Jeon, W., Ramadan, A., Whitall, J., Alissa, N. & Westlake, K. Age-related differences in lower limb muscle activation patterns and balance control strategies while walking over a compliant surface. *Sci. Rep.* **13**, 16555 (2023). 37783842 10.1038/s41598-023-43728-0 PMC10545684

[44] Wunderlich, A., Wollesen, B., Asamoah, J., Delbaere, K. & Li, K. The impact of cognitive-motor interference on balance and gait in hearing-impaired older adults: a systematic review. *Eur. Rev. Aging Phys. Act.* **21**, 17 (2024). 38914940 10.1186/s11556-024-00350-x PMC11194914

[45] Montoya, A. K. Selecting a within- or between-subject design for mediation: validity, causality, and statistical power. *Multivar. Behav. Res.* **58**, 616–636 (2023). 10.1080/00273171.2022.2077287 35679239

[46] Curtze, C., Buurke, T. J. W. & McCrum, C. Notes on the margin of stability. *J. Biomech.* **166**, 112045 (2024). 38484652 10.1016/j.jbiomech.2024.112045

[47] Watson, F. et al. Use of the margin of stability to quantify stability in pathologic gait—a qualitative systematic review. *BMC Musculoskelet. Disord.* **22**, 597 (2021). 34182955 10.1186/s12891-021-04466-4 PMC8240253

[48] Cudejko, T. Effects of footwear on gait biomechanics in people with a history of falls: MATLAB custom codes (figshare, 2025).

