# Supplementary material for: Biomechanical mechanisms underlying the effect of minimalist footwear on walking stability in persons with a history of falls
Source: Commun Med (Lond). 2025 Dec 16;6:39. doi: 10.1038/s43856-025-01291-x (PMC12820056; doi:10.1038/s43856-025-01291-x)
Supplement: Supplementary file 1 — Supplementary Material [file 43856_2025_1291_MOESM1_ESM.pdf]

SUPPLEMENTARY MATERIAL

Biomechanical mechanisms underlying the effect of minimalist footwear on walking stability in persons with a history of falls.

Tomasz Cudejko [1,] *, Asangaedem Akpan [2 3 4], Kristiaan D'Août [5]

[1] Department of Sport, Exercise and Rehabilitation, Northumbria University, Newcastle upon Tune, United Kingdom.
[2] Bunbury Regional Hospital, Western Australia Country Health Service South West, Bunbury, Western Australia, Australia.
[3] Division of Internal Medicine, University of Western Australia, Western Australia, Australia.
[4] Medical School, Faculty of Health, Curtin University, Western Australia, Australia.
[5] Department of Musculoskeletal and Ageing Science, Institute of Life Course and Medical Sciences, University of Liverpool, Liverpool, United Kingdom.
*Corresponding author: E-mail: tomasz.cudejko@northumbria.ac.uk (TC)

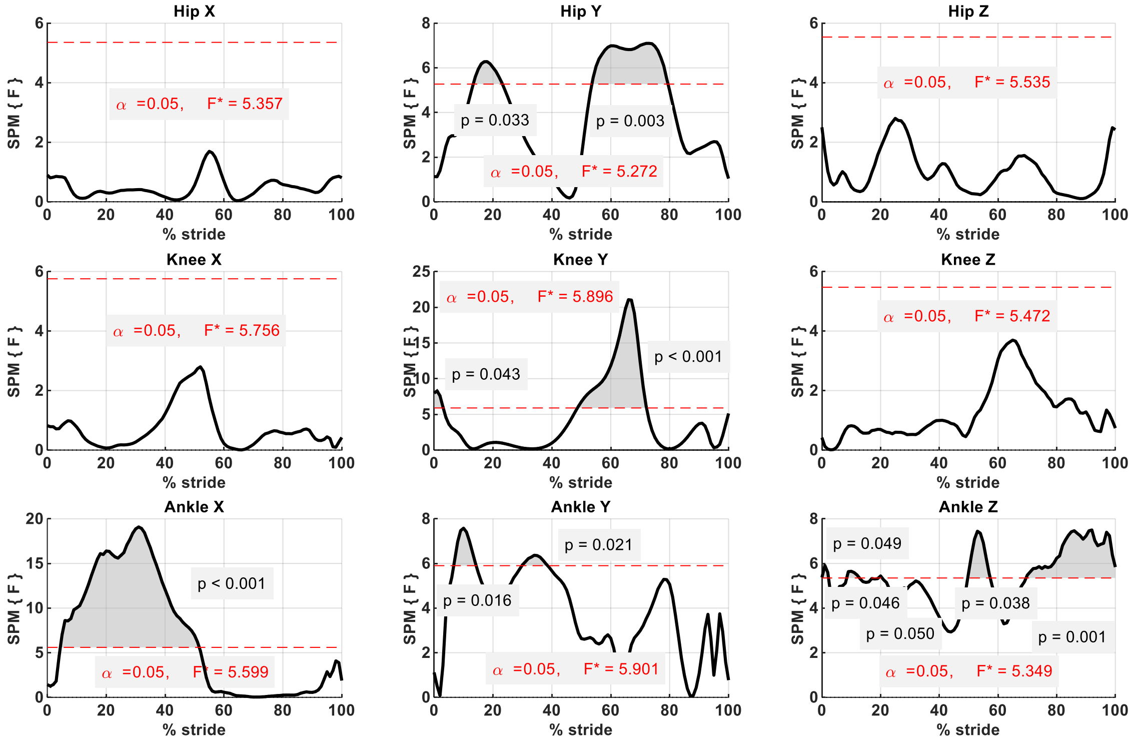

Figure S1: 1 D – SPM, repeated measures ANOVA showing areas of significant differences in joint kinematics between footwear comparisons; Each plot depicts the SPM{F} trajectory across the normalized gait cycle (0–100%) for hip, knee, and ankle joint angles in the frontal (X), sagittal (Y) and transverse (Z) planes. The red dotted line represents the critical threshold (t-critical) for statistical significance at p < 0.05, corrected for multiple comparisons. Regions where the SPM trajectory exceeds this threshold indicate time intervals within the gait cycle where significant differences between footwear conditions were detected

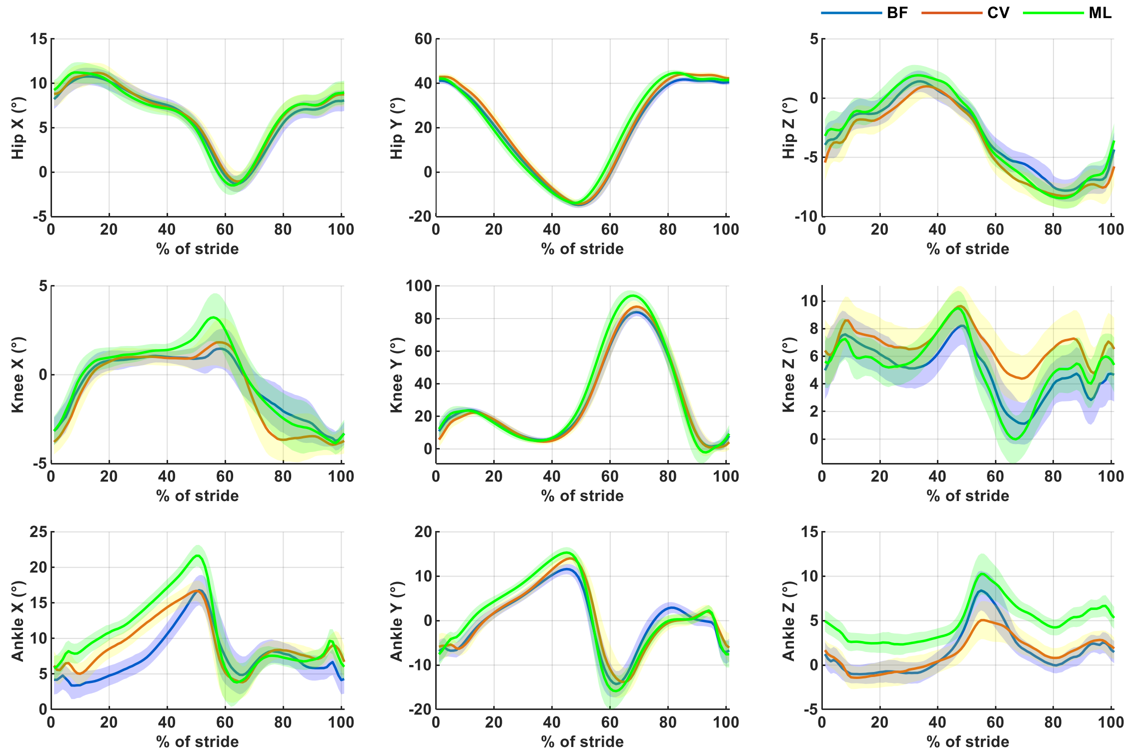

Figure S2. Mean (SD) joint kinematics waveforms (averaged single and dual task) per footwear condition; Plots display ensemble-averaged hip, knee, and ankle joint angle trajectories across the normalized gait cycle (0–100%), where 0% corresponds to heel strike and 100% to the subsequent heel strike of the same limb. Shaded areas represent ±2 standard deviation across participants. BF – Barefoot; CV – Conventional supportive shoe; ML – Minimalist shoe;

Table S1: Number of strides from which joint kinematics were estimated from (strides from left and right limb combined), for each participant stratified by footwear/walking condition

ID	BF ST	BF DT	CV ST	CV DT	ML ST	ML DT
01	30	30	26	25	27	24
02	29	20	29	27	27	25
03	19	23	17	22	27	27
05	30	24	22	23	20	28
06	27	27	25	26	26	27
07	36	37	44	49	58	58
08	30	31	29	27	29	27
09	27	33	26	23	23	37
10	26	31	28	25	29	29
11	35	36	29	30	31	32
12	26	21	21	16	19	17
13	18	26	20	27	20	27
14	19	13	15	19	20	21
15	24	11	13	11	17	16
16	2	2	11	15	9	17
17	23	22	18	19	21	20
18	14	14	16	13	17	16
19	30	30	26	25	27	24
20	29	20	29	27	27	25
21	19	23	17	22	27	27
23	30	24	22	23	20	28
24	27	27	25	26	26	27
25	38	35	42	47	53	56
26	30	31	29	27	29	27
27	27	33	26	23	23	37
29	26	31	28	25	29	29

Abbreviations: ID – participant's ID number; BF – Barefoot; CV – Conventional supportive shoe; ML – Minimalist shoe; ST – Single Task; DT – Dual Task

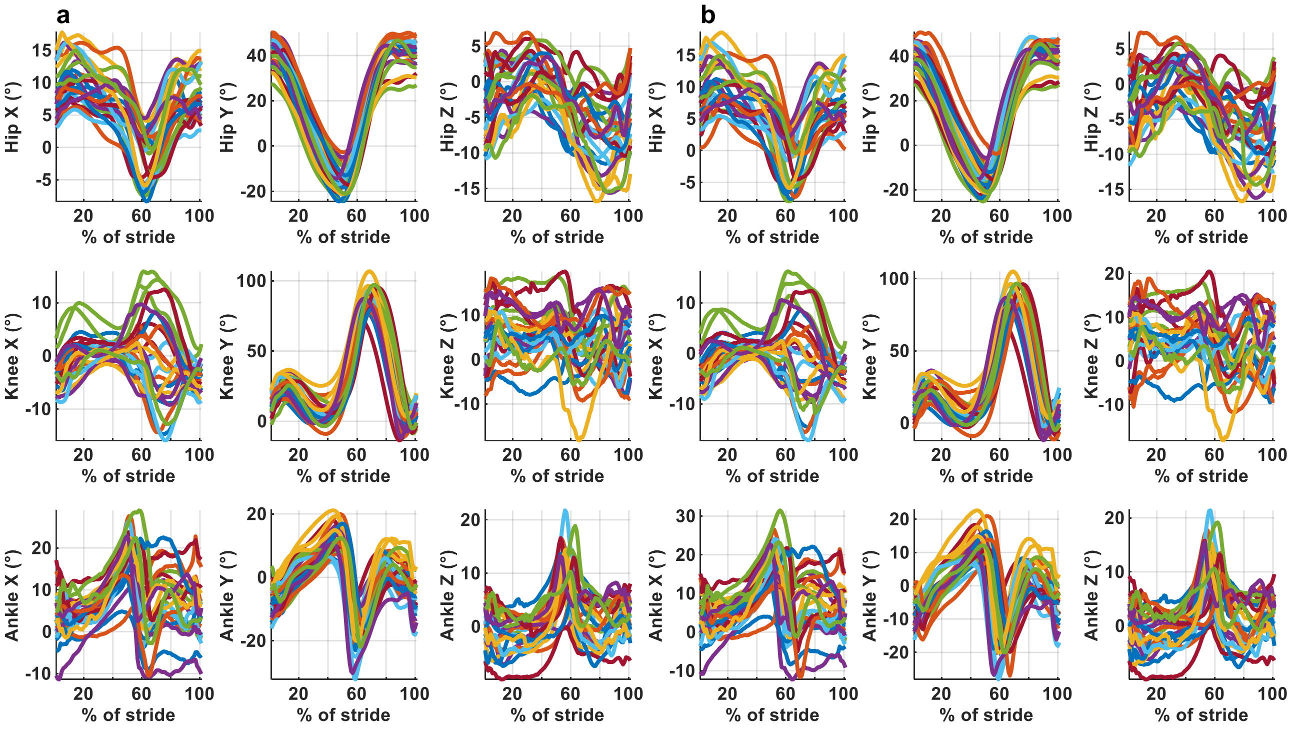

Figure S3: Individual participants' mean joint *kinematics* waveforms while walking barefoot during; a: single-task, and b: dual-task; Waveforms represent mean joint angle trajectories (hip, knee, and ankle) averaged across all walking trials for each participant. Data are time-normalized to 100% of the gait cycle, where 0% corresponds to heel strike and 100% to the subsequent heel strike of the same limb; Abbreviations: X – frontal plane (+/- abduction/ adduction); Y – sagittal plane (+/- flexion/extension); Z – transverse plane (+/- external/internal rotation)

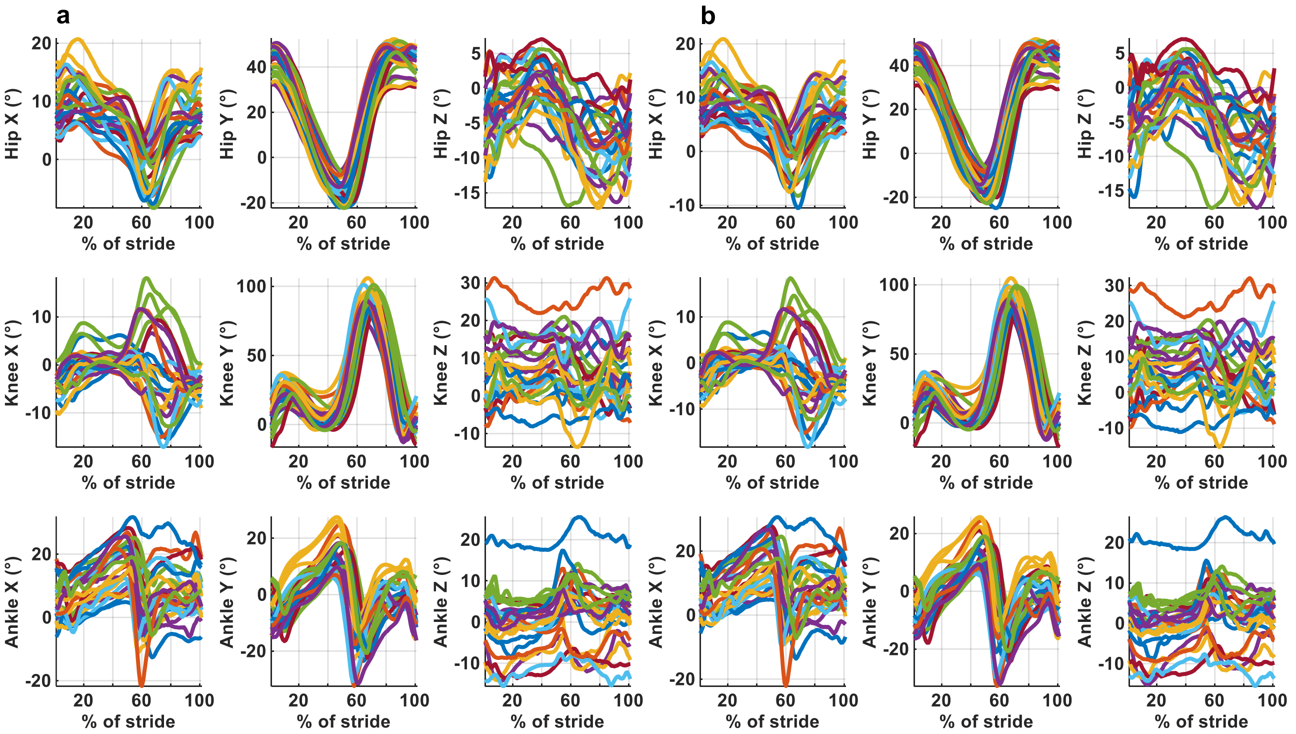

Figure S4: Individual participants' mean joint *kinematics* waveforms while walking in <u>conventional supportive</u> shoes during; a: single-task, and b: dual-task; Waveforms represent mean joint angle trajectories (hip, knee, and ankle) averaged across all walking trials for each participant. Data are time-normalized to 100% of the gait cycle, where 0% corresponds to heel strike and 100% to the subsequent heel strike of the same limb; Abbreviations: X – frontal plane (+/- abduction/ adduction); Y – sagittal plane (+/- flexion/extension); Z – transverse plane (+/- external/internal rotation)

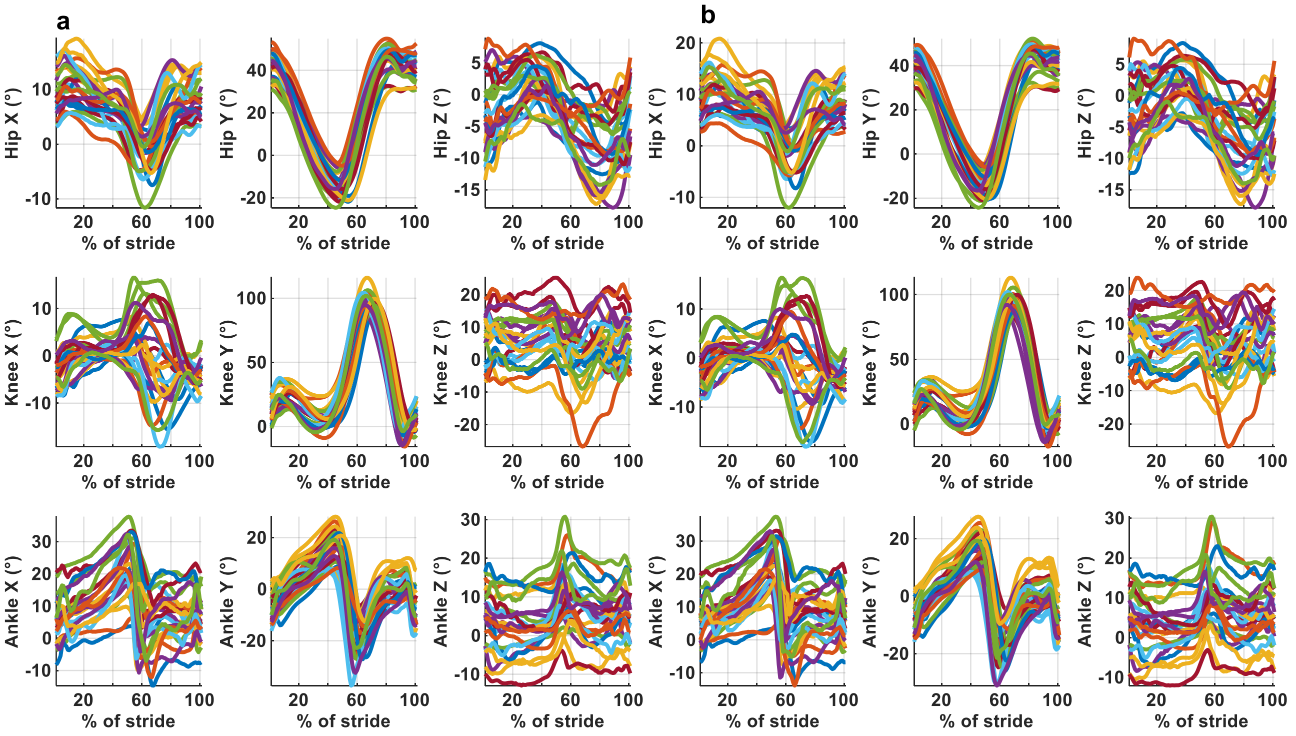

Figure S5: Individual participants' mean joint *kinematics* waveforms while walking in <u>minimalist</u> shoes during; a: single-task, and b: dual-task; Waveforms represent mean joint angle trajectories (hip, knee, and ankle) averaged across all walking trials for each participant. Data are time-normalized to 100% of the gait cycle, where 0% corresponds to heel strike and 100% to the subsequent heel strike of the same limb; Abbreviations: X – frontal plane (+/- abduction/ adduction); Y – sagittal plane (+/- flexion/extension); Z – transverse plane (+/- external/internal rotation)

Table S2. The results of the linear mixed-effect models on the values of kinematics at heel strike for each joint and plane of movement

Hip[1]	Frontal (x)				Sagittal (y)				Transverse (z)			
Pairwise comparisons	Estimate*	df	*p*	95% CIs	Estimate*	df	*p*	95% CIs	Estimate*	df	*p*	95% CIs
Footwear: BF vs CV	-.332	122.330	.251	-.794 .130	**-1.339**	**122.959**	**<.001**	**-2.140 .539**	**1.875**	**124.384**	**<.001**	**1.019 2.732**
Footwear: BF vs ML	**-.772**	**121.003**	**<.001**	**-1.198 .345**	-.615	121.275	.137	-1.354 .125	-.656	121.895	.140	-1.449 .137
Footwear: ML vs CV	**.440**	**120.557**	**.046**	**.006 .873**	-.725	120.711	.063	-1.477 .027	**2.532**	**121.069**	**<.001**	**1.725 3.339**
Interaction effects	Num. df	Den. df	*F*	*p*	Num. df	Den. df	*F*	*p*	Num. df	Den. df	*F*	*p*
Footwear*Walking condition	2	120.044	.125	.882	2	120.056	.625	.537	2	120.083	.061	.941
Footwear*Sex	2	120.245	.140	.869	2	120.315	.955	.388	**2**	**120.482**	**3.904**	**.023**
Knee[1]	Frontal (x)				Sagittal (y)				Transverse (z)			
Pairwise comparisons	Estimate*	df	*p*	95% CIs	Estimate*	df	*p*	95% CIs	Estimate*	df	*p*	95% CIs
Footwear: BF vs CV	**.919**	**124.355**	**<.001**	**.458 1.380**	**7.456**	**130.546**	**<.001**	**5.079 9.832**	**-2.076**	**126.289**	**.008**	**-3.729 .424**
Footwear: BF vs ML	.146	121.883	1.000	-.281 .573	-1.010	124.689	.821	-3.239 1.219	-.645	122.734	.930	-2.180 .890
Footwear: ML vs CV	**.773**	**121.062**	**<.001**	**.339 1.207**	**8.465**	**122.924**	**<.001**	**6.188 10.743**	-1.432	121.574	.084	-2.995 .132
Interaction effects	Num. df	Den. df	*F*	*p*	Num. df	Den. df	*F*	*p*	Num. df	Den. df	*F*	*p*
Footwear*Walking condition	2	120.083	.035	.966	2	120.199	.470	.626	2	120.119	.021	.979
Footwear* Sex	**2**	**120.478**	**12.764**	**<.001**	2	121.489	2.338	.101	2	120.728	.285	.753
Ankle[1,2]	Frontal (x)				Sagittal (y)				Transverse (z)			
Pairwise comparisons	Estimate*	df	*p*	95% CIs	Estimate*	df	*p*	95% CIs	Estimate*	df	*p*	95% CIs
Footwear: BF vs CV	2.587	114.442	.792	-3.015 8.189	-1.055	129.998	1.000	-6.001 3.891	**6.656**	**112.643**	**.003**	**1.836 11.477**
Footwear: BF vs ML	3.960	111.294	.393	-2.366 10.285	1.134	128.415	1.000	-4.460 6.727	5.255	109.308	.062	-.187 10.697
Footwear: ML vs CV	-1.372	134	.407	-3.589 .844	**-2.188**	**133.831**	**.015**	**-4.047 -.330**	1.402	134	.236	-.517 3.320
Interaction effects	Num. df	Den. df	*F*	*p*	Num. df	Den. df	*F*	*p*	Num. df	Den. df	*F*	*p*
Footwear*Walking condition	2	120.269	.044	.957	2	119.291	.074	.929	2	120.270	.064	.938
Footwear* Sex	2	132.363	.230	.794	2	130.399	2.271	.107	2	132.479	2.535	.083

Statistical significance was assessed with two-sided paired-samples t-tests with Bonferroni corrections; Significant effects are shown in bold; Abbreviations: BF – barefoot; CV – conventional supportive shoes; ML – minimalist shoes; [1] model adjusted for walking speed; [2] model adjusted for walking speed, foot length and foot width; * - mean difference; df- degrees of freedom; p – alpha; 95% CIs – 95% Confidence Intervals: Lower Band; Upper Band; BF vs CV – mean range value of the outcome during walking barefoot minus mean range value of the outcome during walking in supportive shoes;

Summary interpretation:

Significant main effects of footwear were observed for each joint and plane of movement (bold text; except ankle frontal plane). No significant interactions were found between footwear and walking condition for any joint or plane of motion. Interactions between footwear and sex were significant for hip (transverse plane) and knee kinematics (frontal plane), indicating sex-related differences in these effects.

Table S3. The results of the linear mixed-effect models on the range values of kinematics for each joint and plane of movement

Hip[1]	Frontal (x)				Sagittal (y)				Transverse (z)			
Pairwise comparisons	Estimate*	df	*p*	95% CIs	Estimate*	df	*p*	95% CIs	Estimate*	df	*p*	95% CIs
Footwear: BF vs CV	-.316	124.061	.535	-.883 .251	**-1.428**	**124.547**	**<.001**	**-2.317 .539**	**-.668**	**124.925**	**.013**	**-1.228 .108**
Footwear: BF vs ML	-.472	121.754	.092	-.996 .052	**-1.346**	**121.966**	**<.001**	**-2.170 .523**	**-.884**	**121.636**	**<.001**	**-1.404 .365**
Footwear: ML vs CV	.156	120.987	1.000	-.378 .689	-.082	121.111	1.000	-.920 .756	.216	120.557	.968	-.312 .745
Interaction effects	Num. df	Den. df	*F*	*p*	Num. df	Den. df	*F*	*p*	Num. df	Den. df	*F*	*p*
Footwear*Walking condition	2	120.077	.487	.616	2	120.086	1.346	.264	2	119.230	1.032	.359
Footwear* Sex	2	120.443	1.587	.209	2	120.502	2.311	.104	2	119.779	.650	.524
Knee[1]	Frontal (x)				Sagittal (y)				Transverse (z)			
Pairwise comparisons	Estimate*	df	*p*	95% CIs	Estimate*	df	*p*	95% CIs	Estimate*	df	*p*	95% CIs
Footwear: BF vs CV	**-1.140**	**124.790**	**.003**	**-1.966 .315**	**-5.312**	**127.719**	**<.001**	**-7.374 3.249**	**-3.572**	**128.739**	**<.001**	**-5.681 1.462**
Footwear: BF vs ML	**-1.613**	**122.073**	**<.001**	**-2.377 .849**	**-10.648**	**122.149**	**<.001**	**-12.576 8.720**	**-2.477**	**123.839**	**.008**	**-4.446 .508**
Footwear: ML vs CV	.473	121.174	.429	-.306 1.251	**5.337**	**120.417**	**<.001**	**3.365 7.305**	-1.095	122.292	.565	-3.104 .914
Interaction effects	Num. df	Den. df	*F*	*p*	Num. df	Den. df	*F*	*p*	Num. df	Den. df	*F*	*p*
Footwear*Walking condition	2	120.091	.339	.713	2	117.976	.770	.465	2	120.165	.086	.918
Footwear* Sex	2	120.532	1.661	.194	2	119.078	1.544	.218	**2**	**121.111**	**3.562**	**.031**
Ankle[1,2]	Frontal (x)				Sagittal (y)				Transverse (z)			
Pairwise comparisons	Estimate*	df	*p*	95% CIs	Estimate*	df	*p*	95% CIs	Estimate*	df	*p*	95% CIs
Footwear: BF vs CV	1.565	133.063	1.000	-5.431 8.562	-1.879	134	1.000	**-6.724 2.966**	**9.554**	**87.884**	**.001**	**3.205 15.902**
Footwear: BF vs ML	-2.432	131.767	1.000	-10.347 5.482	-4.230	134	.191	-9.713 1.252	6.327	82.068	.100	-.812 13.466
Footwear: ML vs CV	**3.998**	**133.915**	**<.001**	**1.389 6.607**	**2.351**	**132.092**	**.005**	**.583 4.119**	**3.227**	**134**	**.015**	**.481 5.973**
Interaction effects	Num. df	Den. df	*F*	*p*	Num. df	Den. df	*F*	*p*	Num. df	Den. df	*F*	*p*
Footwear*Walking condition	2	120.244	.109	.897	2	119.201	1.654	.196	2	117.373	.170	.844
Footwear* Sex	2	130.728	1.902	.153	2	129.152	1.700	.187	**2**	**131.963**	**3.481**	**.034**

Statistical significance was assessed with two-sided paired-samples t-tests with Bonferroni correction; Significant effects are shown in bold; Abbreviations: BF – barefoot; CV – conventional supportive shoes; ML – minimalist shoes; [1] model adjusted for walking speed; [2] model adjusted for foot length and foot width; * - mean difference; df- degrees of freedom; p – alpha; 95% CIs – 95% Confidence Intervals: Lower Band; Upper Band; BF vs CV – mean range value of the outcome during walking barefoot minus mean range value of the outcome during walking in supportive shoes;

Summary interpretation:

Significant main effects of footwear were observed for each joint and plane of movement (bold text; except hip frontal plane). No significant interactions were found between footwear and walking condition for any joint or plane of motion. Interactions between footwear and sex were significant for knee and ankle kinematics (transverse plane) and knee kinematics (frontal plane), indicating sex-related differences in these effects.

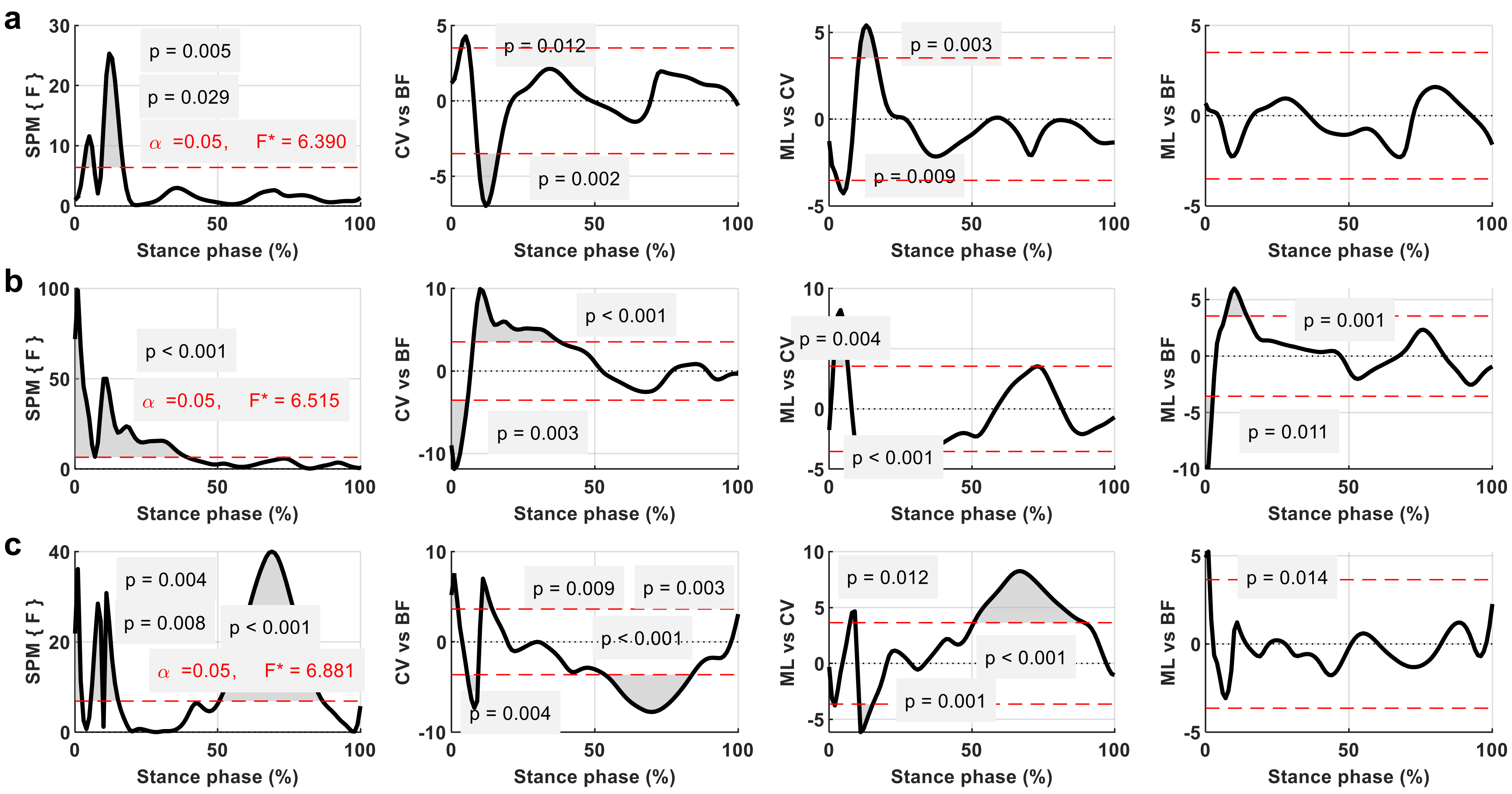

Figure S6: 1 D – SPM, repeated measures ANOVA showing areas of significant differences in a. hip, b. knee and c. ankle powers between footwear comparisons; Each plot depicts the SPM{F} trajectory across the normalized stance phase (0–100%) for hip, knee, and ankle joint powers. The red dotted line represents the critical threshold (t-critical) for statistical significance at p < 0.05, corrected for multiple comparisons. Regions where the SPM trajectory exceeds this threshold indicate time intervals within the stance phase where significant differences between footwear conditions were detected Abbreviations: BF – Barefoot; CV – Conventional supportive shoe; ML – Minimalist shoe

.

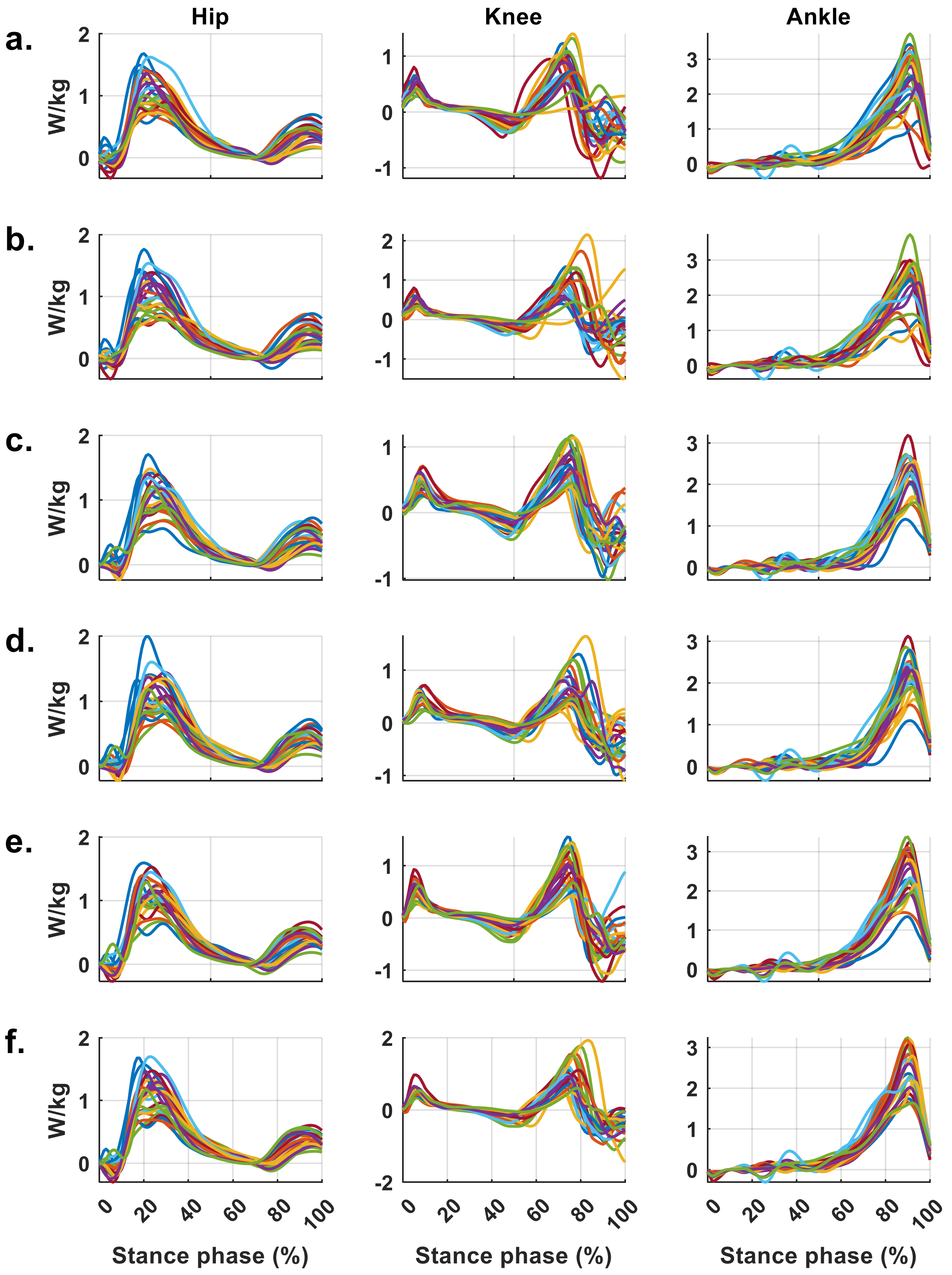

Figure S7. Individual participants' mean hip, knee, and ankle joint power waveforms across footwear and task conditions. Each subplot shows mean joint power trajectories averaged across all walking trials for individual participants under the following conditions: (a) barefoot single-task, (b) barefoot dual-task, (c) conventional supportive shoes single-task, (d) conventional supportive shoes dual-task, (e) minimalist shoes single-task, and (f) minimalist shoes dual-task. Joint powers were computed from synchronized 3D kinematic and ground reaction force data and normalized to body mass (expressed in W/kg). Data are time-normalized to 100% of the gait cycle, where 0% corresponds to heel strike and 100% to the toe-off. Positive values indicate power generation (concentric muscle action), while negative values represent power absorption (eccentric muscle action); Abbreviations W/kg – Watts per kilogram of body weight:

Table S4: Number of steps (n of correct steps on force plates) from which joint kinetics were estimated from, for each participant stratified by footwear/walking condition

ID	BF ST	BF DT	CV ST	CV DT	ML ST	ML DT
01	20	8	19	11	16	11
02	13	12	11	9	2	4
03	5	4	8	8	17	17
05	20	12	10	6	16	23
06	16	17	9	5	2	14
07	14	11	11	15	3	12
08	6	6	10	10	5	8
09	9	16	13	8	13	10
10	18	4	13	15	6	12
11	5	8	10	6	8	8
12	14	11	15	14	6	7
13	3	3	7	10	4	13
14	17	7	8	8	10	9
15	7	7	15	12	5	10
16	9	12	2	12	5	3
17	8	9	3	2	16	14
18	18	15	17	14	6	15
19	13	12	14	12	16	17
20	17	18	13	12	11	12
21	11	9	6	8	8	6
23	12	14	4	4	13	14
24	10	7	6	7	7	8
25	8	1	7	7	11	15
26	5	1	7	8	7	15
27	9	12	6	5	9	6
29	8	2	2	9	12	14

Abbreviations: ID – participant’s ID number; BF – Barefoot; CV – Conventional supportive shoe; ML – Minimalist shoe; ST – Single Task; DT – Dual Task

Table S5. The results of the linear mixed-effect models on the range, peak positive and peak negative values of hip, knee and ankle powers

Hip[1]	Range				Peak positive				Peak negative			
Pairwise comparisons	Estimate*	df	*p*	95% CIs	Estimate*	df	*p*	95% CIs	Estimate*	df	*p*	95% CIs
Footwear: BF vs CV	.002	120.800	1.000	-.062 .066	-.020	120.501	1.000	-.072 .033	-.021	122.537	.070	-.044 .001
Footwear: BF vs ML	**-.070**	**120.560**	**.022**	**-.133 .008**	**-.054**	**120.332**	**.036**	-.105 .003	.017	121.911	.187	-.005 .039
Footwear: ML vs CV	**.072**	**120.313**	**.019**	**.009 .135**	.034	120.154	.328	-.017 .086	**-.038**	**121.362**	**<.001**	-.061 -.016
Interaction effects	Num. df	Den. df	*F*	*p*	Num. df	Den. df	*F*	*p*	Num. df	Den. df	*F*	*p*
Footwear*Walking condition	2	119.883	2.756	.068	2	119.854	2.636	.076	2	120.187	.918	.402
Footwear*Sex	**2**	**119.837**	**3.349**	**.038**	2	119.822	2.896	.059	2	120.070	1.936	.149
Knee[1]	Range				Peak positive				Peak negative			
Pairwise comparisons	Estimate*	df	*p*	95% CIs	Estimate*	df	*p*	95% CIs	Estimate*	df	*p*	95% CIs
Footwear: BF vs CV	.086	122.408	.478	-.061 .233	.071	122.002	.171	-.019 .161	-.015	124.233	1.000	-.098 .067
Footwear: BF vs ML	-.114	121.814	.174	-.258 .031	**-.102**	**121.511**	**.017**	**-.190 -.014**	.011	123.169	1.000	-.070 .092
Footwear: ML vs CV	**.200**	**121.287**	**.004**	**.054 .346**	**.173**	**121.054**	**<.001**	**.085 .262**	-.026	122.433	1.000	-.108 .056
Interaction effects	Num. df	Den. df	*F*	*p*	Num. df	Den. df	*F*	*p*	Num. df	Den. df	*F*	*p*
Footwear*Walking condition	2	120.177	.590	.556	2	120.146	.363	.696	2	120.320	.671	.513
Footwear*Sex	2	120.066	.588	.557	2	120.054	.042	.959	2	120.131	1.620	.202
Ankle[1]	Range				Peak positive				Peak negative			
Pairwise comparisons	Estimate*	df	*p*	95% CIs	Estimate*	df	*p*	95% CIs	Estimate*	df	*p*	95% CIs
Footwear: BF vs CV	**.347**	**121.164**	**<.001**	**.220 .474**	**.303**	**121.128**	**<.001**	**.181 .426**	**-.043**	**121.090**	**<.001**	**-.057 .029**
Footwear: BF vs ML	-.035	120.881	1.000	-.160 .089	-.057	120.853	.752	-.177 .063	**-.021**	**120.825**	**<.001**	**-.035 .008**
Footwear: ML vs CV	**.382**	**120.595**	**<.001**	**.257 .508**	**.360**	**120.575**	**<.001**	**.239 .482**	**-.022**	**120.555**	**<.001**	**-.036 .008**
Interaction effects	Num. df	Den. df	*F*	*p*	Num. df	Den. df	*F*	*p*	Num. df	Den. df	*F*	*p*
Footwear*Walking condition	2	120.085	.940	.394	2	120.082	.670	.513	2	120.079	2.661	.074
Footwear*Sex	2	120.030	.758	.471	2	120.029	.835	.436	2	120.028	2.043	.134

Statistical significance was assessed with two-sided paired-samples t-tests with Bonferroni corrections; Significant effects are shown in bold; Abbreviations: BF – barefoot; CV – conventional supportive shoes; ML – minimalist shoes; [1] model adjusted for walking speed; * - mean difference; df- degrees of freedom; p – alpha; 95% CIs – 95% Confidence Intervals: Lower Band; Upper Band; BF vs CV – mean range value of the outcome during walking barefoot minus mean range value of the outcome during walking in supportive shoes;

Summary interpretation:

Significant main effects of footwear were observed for each joint power (bold text; except knee power absorption). No significant interactions were found between footwear and walking condition for any joint or plane of motion. Interactions between footwear and sex were significant for hip power generation (range values), indicating sex-related differences in this effect.

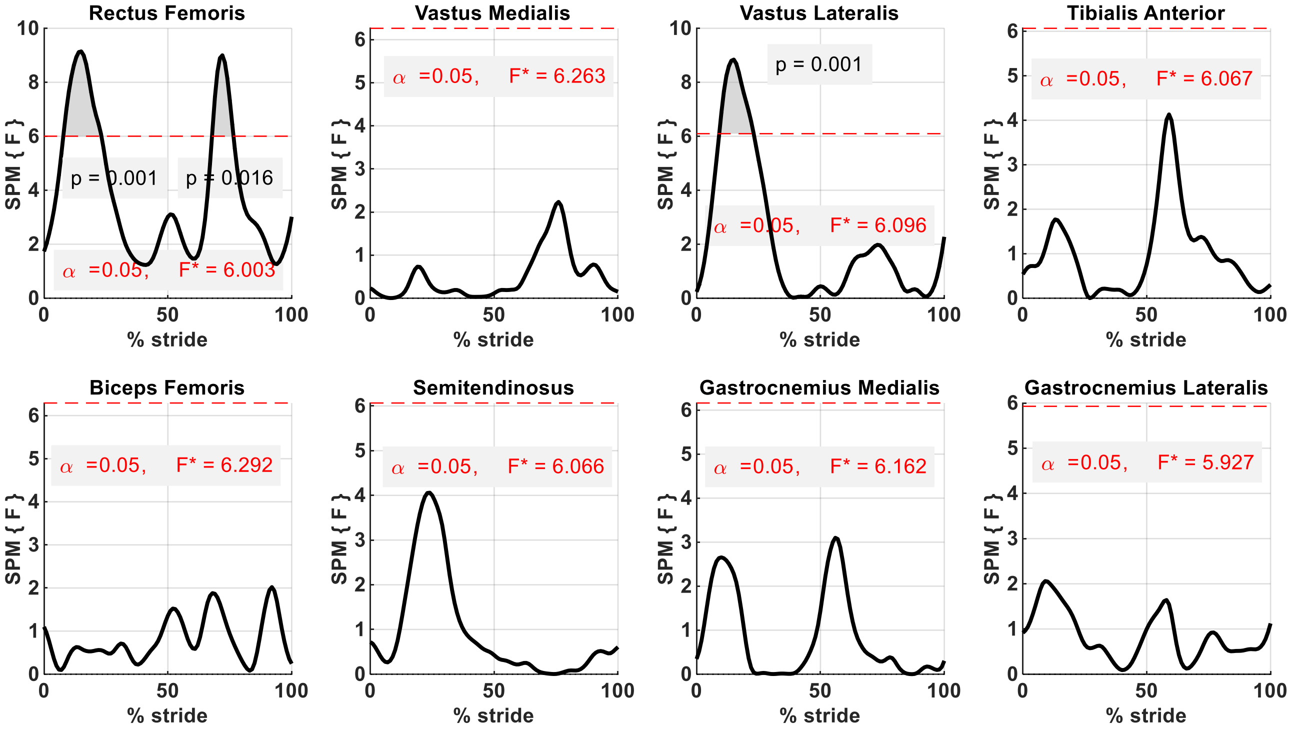

Figure S8: 1 D – SPM, repeated measures ANOVA showing areas of significant differences in muscle activity between footwear comparisons; Each plot displays the SPM{t} trajectory across the normalized gait cycle (0–100%) for individual lower-limb muscles: rectus femoris, vastus lateralis, vastus medialis, tibialis anterior, biceps femoris, semitendinosus, gastrocnemius lateralis, and gastrocnemius medialis. The red dotted line indicates the critical threshold (t-critical) for statistical significance at $p < 0.05$, corrected for multiple comparisons. Segments where the SPM{t} trajectory exceeds this threshold represent time intervals within the gait cycle during which significant differences in EMG amplitude occurred between footwear conditions

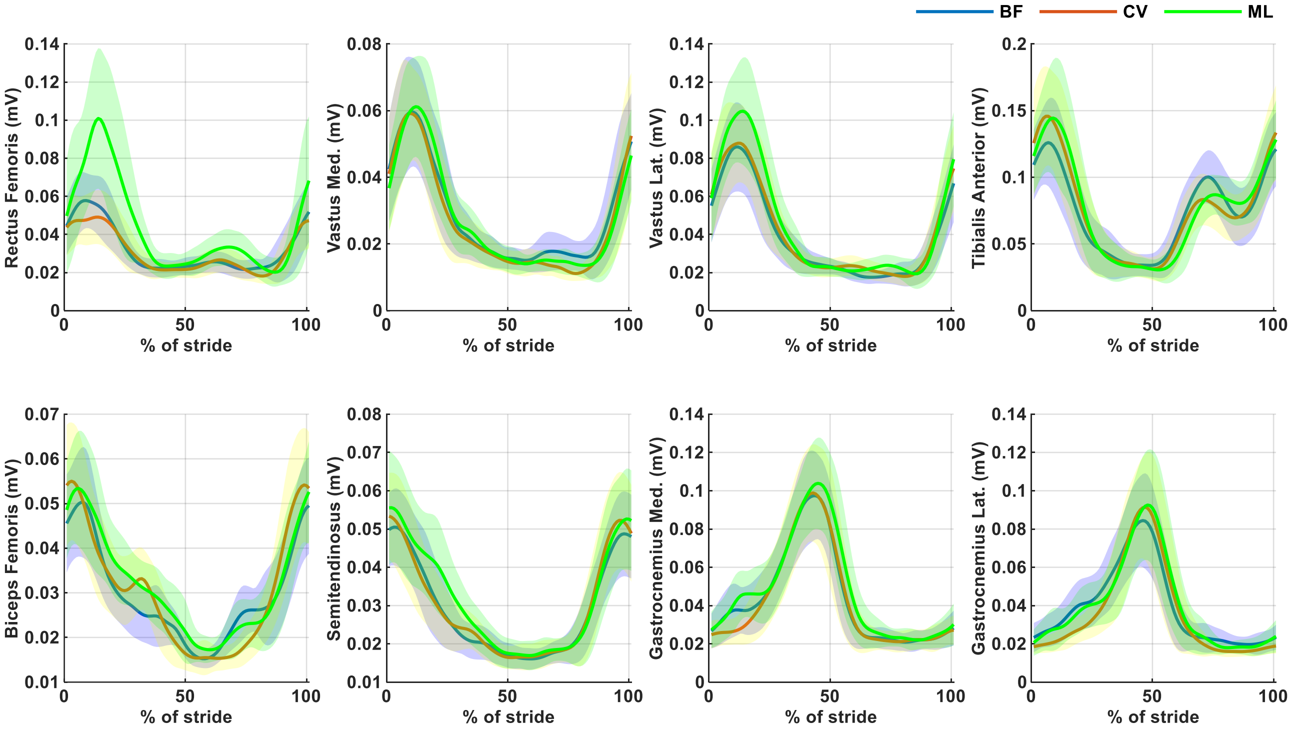

Figure S9. Mean (SD) EMG waveforms (averaged single and dual task) per footwear condition; Plots display ensemble-averaged rectified and time-normalized EMG signals across the gait cycle (0–100%, heel strike to subsequent heel strike). Data are shown for three footwear conditions: barefoot (BF), conventional supportive shoes (CV), and minimalist shoes (ML). Traces represent mean activation patterns for eight lower-limb muscles: rectus femoris, vastus lateralis (Lat.), vastus medialis (Med.), tibialis anterior, biceps femoris, semitendinosus, gastrocnemius lateralis (Lat.), and gastrocnemius medialis (Med.) Shaded regions indicate ±1 standard deviation across participants. Abbreviations: mV – millivolts; Med – Medialis; Lat. - Lateralis

Table S6: Number of strides from which EMG data was obtained from, for each participant stratified by footwear/walking condition

ID	BF ST	BF DT	CV ST	CV DT	ML ST	ML DT
01	24	24	20	17	18	14
05	14	16	14	14	13	16
06	15	15	13	13	12	15
07	15	16	12	12	14	14
08	12	12	14	14	15	13
09	12	14	12	10	12	13
10	17	17	15	18	14	16
11	12	17	12	12	12	12
12	14	11	17	13	12	13
13	12	17	11	13	13	14
14	17	12	11	12	11	13
15	13	13	12	11	11	13
16	12	12	12	12	12	10
17	18	14	12	17	17	15
18	13	10	16	14	14	21
19	11	15	12	10	12	12
20	16	12	14	15	14	17
21	14	13	10	8	7	9
22	8	5	10	6	8	8
23	12	15	10	12	12	15
24	9	6	8	11	9	11
25	12	5	7	6	7	10
26	6	2	10	16	14	16
27	12	12	12	12	12	12
29	7	9	6	6	9	8

Abbreviations: ID – participant's ID number; BF – Barefoot; CV – Conventional shoe; ML – Minimal shoe; ST – Single Task; DT – Dual Task

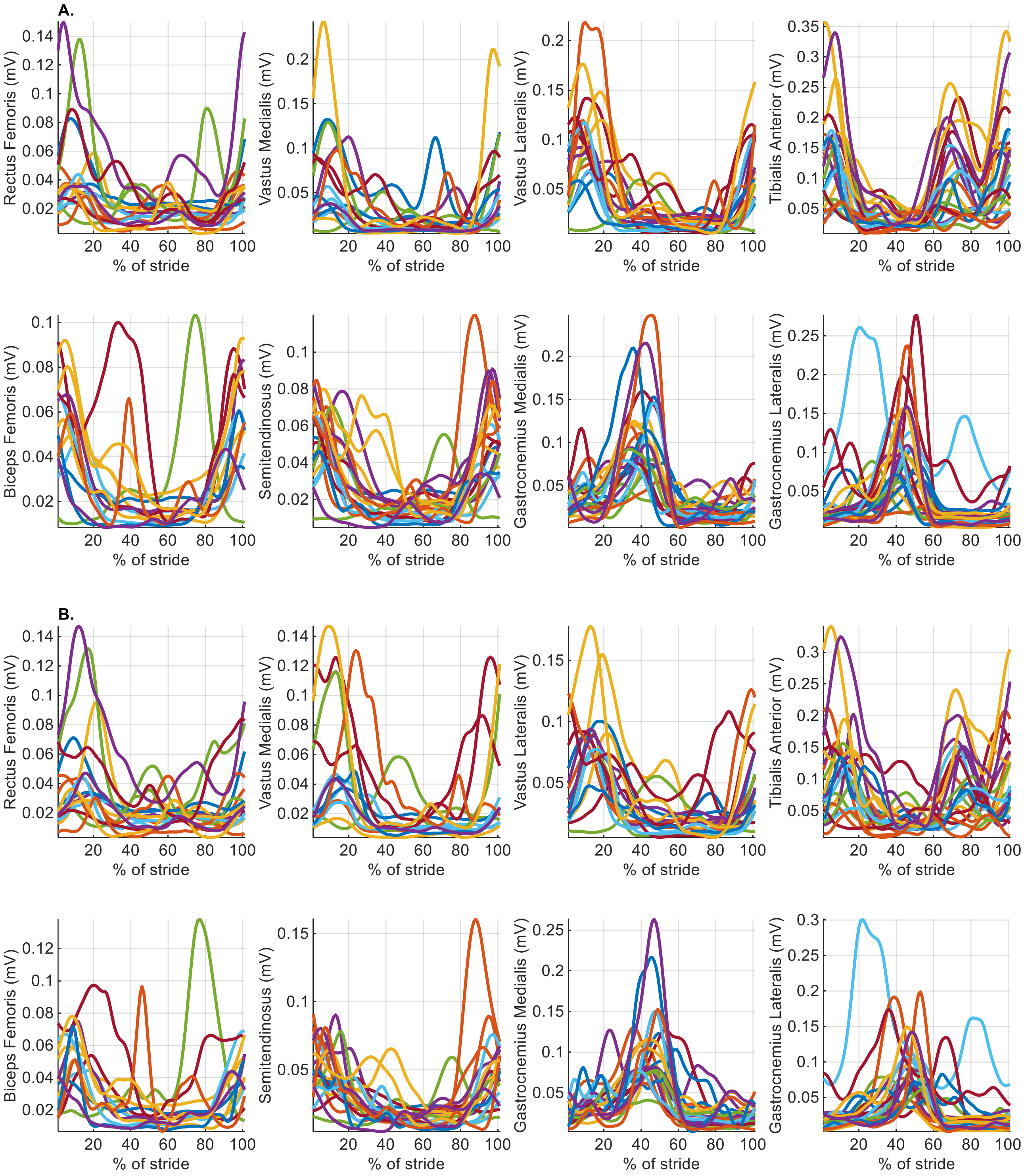

Figure S10: Individual participants' mean *EMG amplitudes* while walking barefoot during; A: single-task, and B: dual-task; Each plot represents the mean rectified and time-normalized EMG signal for individual participants across all walking trials. Data are presented for eight lower-limb muscles: rectus femoris, vastus lateralis, vastus medialis, tibialis anterior, biceps femoris, semitendinosus, gastrocnemius lateralis, and gastrocnemius medialis. The x-axis represents the gait cycle (0–100%, heel strike to subsequent heel strike of the same limb), and the y-axis shows EMG amplitude (mV);.Abbreviations: mV – millivolts.

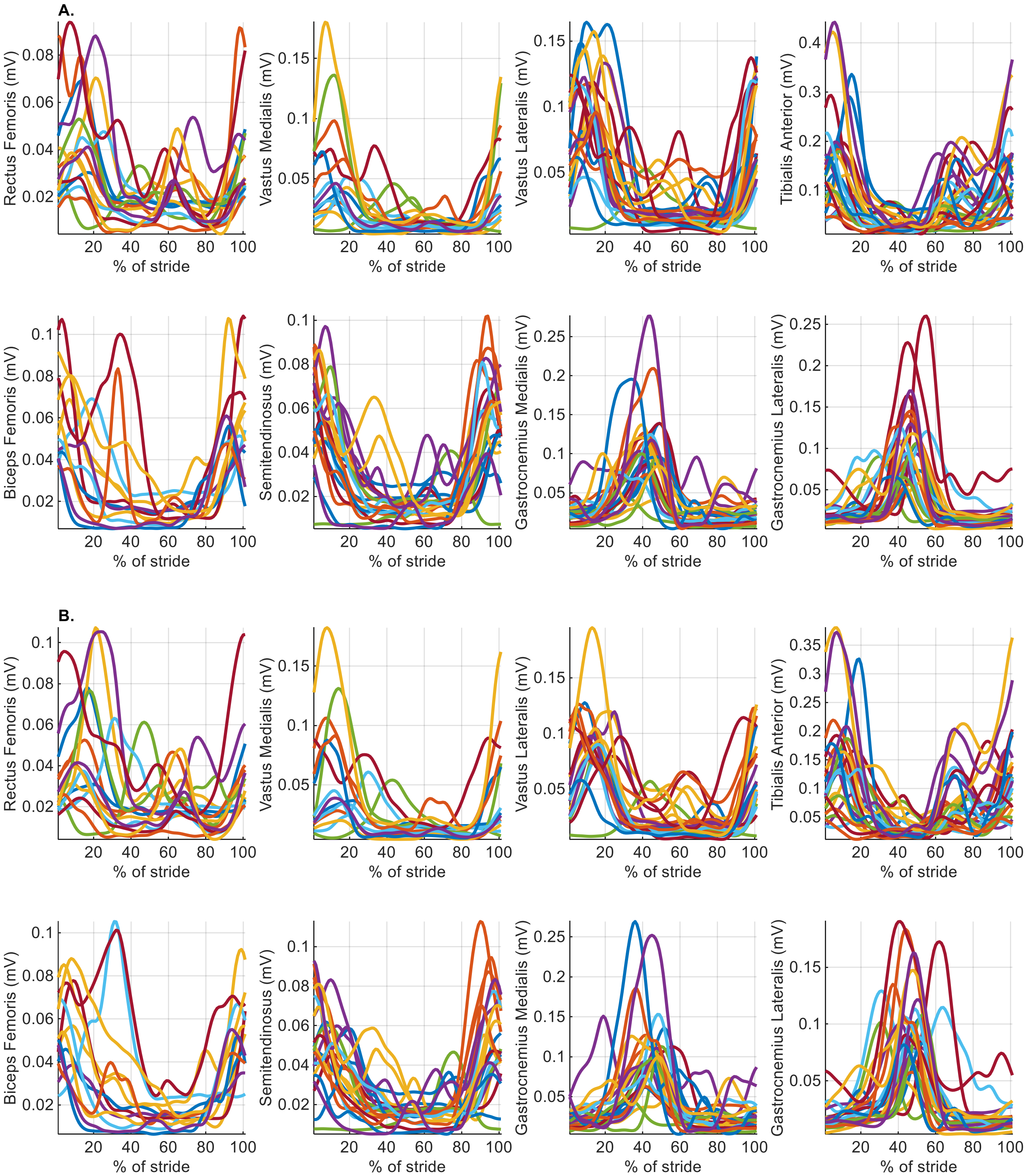

Figure S11: Individual participants' mean *EMG amplitudes* while walking in conventional supportive shoes during; Each plot represents the mean rectified and time-normalized EMG signal for individual participants across all walking trials. Data are presented for eight lower-limb muscles: rectus femoris, vastus lateralis, vastus medialis, tibialis anterior, biceps femoris, semitendinosus, gastrocnemius lateralis, and gastrocnemius medialis. The x-axis represents the gait cycle (0–100%, heel strike to subsequent heel strike of the same limb), and the y-axis shows EMG amplitude (mV).A: single-task, and B: dual-task; Abbreviations: mV – millivolts.

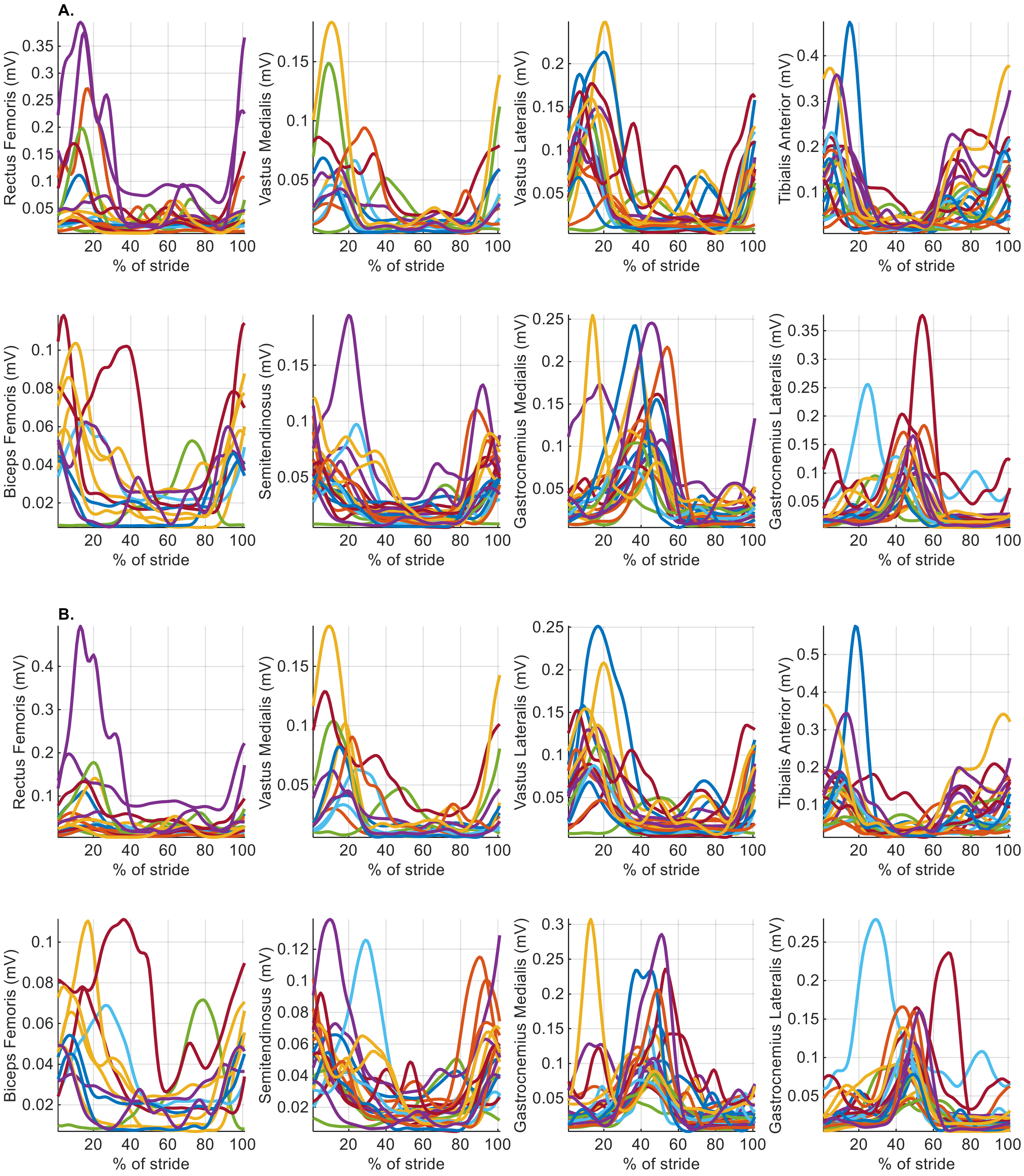

Figure S12: Individual participants' mean *EMG amplitudes* while walking in minimalist shoes during; Each plot represents the mean rectified and time-normalized EMG signal for individual participants across all walking trials. Data are presented for eight lower-limb muscles: rectus femoris, vastus lateralis, vastus medialis, tibialis anterior, biceps femoris, semitendinosus, gastrocnemius lateralis, and gastrocnemius medialis. The x-axis represents the gait cycle (0–100%, heel strike to subsequent heel strike of the same limb), and the y-axis shows EMG amplitude (mV).A: single-task, and B: dual-task; Abbreviations: mV – millivolts.

Table S7. The results of the linear mixed-effect models on the range values of the EMG amplitudes for each lower-limb muscle

	Rectus Femoris[1]				Vastus Medialis[1]				Vastus Lateralis[1]				Tibialis Anterior[1]			
Pairwise comparisons	Estimate	df	*p*	95% CIs	Estimate	df	*p*	95% CIs	Estimate	df	*p*	95% CIs	Estimate	df	*p*	95% CIs
Footwear: BF vs CV	.001	84.5	1.00	-.036 .038	.004	69.6	1.00	-.008 .016	.007	104	.79	-.009 .023	.006	120.5	1.00	-.010 .023
Footwear: BF vs ML	**-.055**	**84.8**	**.001**	**-.092 -.019**	-.005	67.7	.90	-.017 .007	**-.020**	**91.6**	**.005**	**-.036 -.005**	-.005	116.7	1.00	-.021 .011
Footwear: ML vs CV	**.056**	**81.0**	**.001**	**.019 .093**	.009	69.1	.19	-.003 .021	**.028**	**94.5**	**<.001**	**.012 .043**	.012	117.2	.22	-.004 .028
Interaction effects	Num. df	df	*F*	*p*	Num. df	df	*F*	*p*	Num. df	df	*F*	*p*	Num. df	df	*F*	*p*
Footwear*Walk condition	2	76.6	.34	.709	2	66.6	.60	.551	2	87.6	1.05	.352	2	115.7	.44	.646
Footwear*Sex	2	80.7	1.44	.241	2	67.3	.76	.467	2	89.6	.30	.742	2	115.8	1.83	.164
	Biceps Femoris[1]				Semitendinosus[1]				Gastrocnemius Medialis[1]				Gastrocnemius Lateralis[1]			
Pairwise comparisons	Estimate	df	*p*	95% CIs	Estimate	df	*p*	95% CIs	Estimate	df	*p*	95% CIs	Estimate	df	*p*	95% CIs
Footwear: BF vs CV	.003	68	1.00	-.007 .014	.003	116.6	1.00	-.006 .011	.004	116.4	1.00	-.008 .017	.012	125.4	.070	-.001 .025
Footwear: BF vs ML	.002	65.6	1.00	-.009 .012	-.007	111.3	.11	-.015 .001	**-.013**	**113.2**	**.028**	**-.025 -.001**	-.003	121.4	1.00	-.015 .010
Footwear: ML vs CV	.002	66.0	1.00	-.009 .012	**.009**	**110.8**	**.011**	**.002 .017**	**.018**	**113.2**	**.001**	**.006 .029**	**.015**	**121.3**	**.011**	**.003 .027**
Interaction effects	Num. df	df	*F*	*p*	Num. df	df	*F*	*p*	Num. df	df	*F*	*p*	Num. df	df	*F*	*p*
Footwear*Walk condition	2	62.3	.25	.778	2	107.2	.69	.504	2	111.7	.27	.759	2	119.7	.40	.669
Footwear*Sex	2	63.1	.56	.572	**2**	**108.8**	**3.20**	**.045**	2	111.9	.71	.489	**2**	**119.8**	**4.28**	**.016**

Statistical significance was assessed with two-sided paired-samples t-tests with Bonferroni corrections; Significant effects are shown in bold; Abbreviations: BF – barefoot; CV – conventional supportive shoes; ML – minimalist shoes; [1] model adjusted for walking speed; df- degrees of freedom; p – alpha; 95% CIs – 95% Confidence Intervals: Lower Band; Upper Band; BF vs CV – mean range value of the outcome during walking barefoot minus mean range value of the outcome during walking in supportive shoes.

Summary interpretation:

Significant main effects of footwear were observed for rectus femoris, vastus lateralis, semitendinosus and gastrocnemius muscles (bold text). No significant interactions were found between footwear and walking condition for any of the muscles. Interactions between footwear and sex were significant for semitendinosus and gastrocnemius lateralis, indicating sex-related differences in these effects.

Table S8. Comparisons between footwear types for walking speed, foot length, foot width

	BF	CV	ML	BF vs. CV	BF vs ML	ML vs CV
	Mean ± SD	Mean ± SD	Mean ± SD	*p*	*p*	*p*
Walking speed (m/s)	1.37 ± 0.15	1.44 ± 0.14	1.41 ± 0.14	**0.04**	0.56	0.73
Foot length (cm)[1]	21.2 ± 1.44	23.5 ± 2.49	23.9 ± 1.85	**<0.001**	**<0.001**	0.93
Foot width (cm)[2]	8.3 ± 0.75	10.2 ± 0.67	10.5 ± 0.63	**<0.001**	**<0.001**	**0.02**

Abbreviations: BF – barefoot; CV – conventional supportive shoes; ML – minimalist shoe; m/s – meters per second; cm – centimetres; [1] Defined as distance between heel marker and hallux marker; [2] defined as distance between marker on the base of the 5th metatarsal bone and base of the 1st metatarsal bone; Significant differences are shown in bold
