# Supplementary material for: Biomechanical mechanisms underlying the effect of minimalist footwear on walking stability in persons with a history of falls
Source: Commun Med (Lond). 2025 Dec 16;6:39. doi: 10.1038/s43856-025-01291-x (PMC12820056; doi:10.1038/s43856-025-01291-x)
Supplement: Supplementary file 2 — Description of Additional Supplementary Files [file 43856_2025_1291_MOESM2_ESM.pdf]

Description of Additional Supplementary Files

File name: Supplementary Data 1

Description: Source data for Fig. 6a–b
Individual participants' mean (average left and right limb) hip, knee and ankle 3D joint angle waveforms (from heel strike to subsequent heel strike of the same limb) stratified by footwear and walking condition (single task and dual-task).

File name: Supplementary Data 2

Description: Source data for Fig. 6c-f
(i)Anterior–posterior MoS values at heel strike and (ii) hip, knee, and ankle joint angle ranges and heel-strike angles for each participant, stratified by footwear and walking condition (single task and dual-task).

File name: Supplementary Data 3

Description: Source data for Fig. 7a–b
Individual participants' mean (average left and right limb) hip, knee and ankle joint power waveforms (from heel strike to toe off of the same limb) stratified by footwear and walking condition (single task and dual-task).

File name: Supplementary Data 4

Description: Source data for Fig. 7c–e
(i)Anterior–posterior MoS values at heel strike and (ii) hip, knee, and ankle joint power range values for each participant, stratified by footwear and walking condition.

File name: Supplementary Data 5

Description: Source data for Fig. 8a–b
Individual participants' mean EMG waveforms (from heel strike to subsequent heel strike of the 700 same limb) for each muscle stratified by footwear and walking condition (single task and dual-task).

File name: Supplementary Data 6

Description: Source data for Fig. 8c-g

(i) Anterior–posterior MoS values at heel strike and (ii) EMG range values for each muscle and participant, stratified by footwear and walking condition (single task and dual task).
